# Superior synergistic corrosion inhibition of brass in NaCl solution by 2-mercaptobenzothiazole and TiO_2_ nanoparticles compared with SiO_2_

**DOI:** 10.1038/s41598-026-55234-0

**Published:** 2026-06-13

**Authors:** Eman AbdElRhiem, Mohamed Y. Sedek, N. M. Badawy, Mohamed M. Megahed, Saad G. Mohamed

**Affiliations:** 1 Mining and Metallurgy Engineering Department, Tabbin Institute for Metallurgical Studies (TIMS), 109, Tabbin, Helwan, Cairo 11421 Egypt; 2 Conservation Department, Faculty of Archaeology, Fayoum University, Fayoum, Egypt; 3 Faculty of Archaeology and Tourism Guidance, Misr University for Science and Technology, 6th of October City, Egypt; 4 Chemistry Department, Faculty of Engineering, Ain Shams University, Cairo, Egypt; 5 Chemistry Department, Faculty of Science, Al-Baha University, Al Baha, Kingdom of Saudi Arabia

**Keywords:** Brass alloy, 2-Mercaptobenzothiazole (MBT) inhibitor, Silica and titanium dioxide addition, Electrochemical behavior, Density functional theory (DFT), Chemistry, Materials science, Nanoscience and technology

## Abstract

2-Mercaptobenzothiazole (MBT) combined with nanoparticles exhibits a synergistic corrosion inhibition effect for brass in 3.5% NaCl solution. The corrosion behavior was evaluated using open circuit potential (OCP), electrochemical impedance spectroscopy (EIS), and potentiodynamic polarization techniques. The inhibition efficiency increased with MBT concentration (0–20 ppm), reaching 97.4% at 20 ppm. The addition of 10 ppm nanoparticles further enhanced the inhibition performance. In particular, TiO_2_ nanoparticles improved the efficiency to 98.2%, showing superior performance compared with SiO_2_ nanoparticles (94%). The adsorption studies showed that the Langmuir isotherm model (R² = 0.9941) was adopted by MBT, with ΔG°_ads_ = (− 12.66 kJ mol^−1^) and K_ads_ = 2.98 L mol^−1^, indicating spontaneous adsorption dominated by physical with weak chemical interaction. Density Functional Theory (DFT) calculations at the B3LYP/LANL2DZ level were performed to study the electronic properties and adsorption behavior of MBT and its nanoparticle-modified systems. The results show that nanoparticle incorporation enhances the reactivity and adsorption characteristics of MBT. The MBT–TiO_2_ system exhibits the most negative adsorption energy, smaller energy gap, and higher charge transfer ability (ΔN_max_), indicating strong interaction with partial chemisorption character, while the MBT–SiO_2_ system shows weaker adsorption despite higher electrophilicity. This improvement is attributed to the synergistic interaction between MBT and the incorporated nanoparticles, which enhanced the adsorption strength of inhibitor molecules onto the brass surface and promoted the formation of a highly compact protective layer through effective filling of surface pores and structural imperfections. The originality of this work is demonstrated by the fact that the electronic nature of the nanoparticles, rather than the physical barrier effect, dominates the compactness, interfacial stability and electrochemical performance of the MBT-based protective films.

**Supplementary Information:**

The online version contains supplementary material available at 10.1038/s41598-026-55234-0.

## Introduction

Brass is commonly employed in plumbing systems because of its high corrosion resistance and mechanical qualities; nonetheless, it can suffer from many types of corrosion, including galvanic corrosion and dezincification. Dezincification is a hazard for alloys with more than 15% zinc. Dezincification develops a layer of porous copper alloy, on a structural level, affecting the component and causing failure^1^. Corrosion causes failure, which not only results in costly inspection, repair, and replacement costs but also poses a public risk. Controlling brass corrosion has been significant in a variety of businesses, particularly in industry and artifacts^2^. However, it is difficult to totally eliminate corrosion; there are numerous methods for avoiding or reducing the corrosion rate^3^. Corrosion inhibitors are extremely practical since they are widely used in decreasing metallic waste during production and lowering the danger of material failure, both of which can result in the abrupt closure of industrial processes, incurring additional expenditures. Organic compounds have proven to be quite effective at inhibiting aqueous corrosion in a variety of metals and alloys. It was previously proved that molecules that include nitrogen and sulfur^4^ produce stronger inhibition than those containing only one of the atoms at a time, which is due to their molecular structure.

As a result, it is crucial to design unique chemicals that act as corrosion inhibitors. Using a variety of inhibitors is a viable technique for enhancing corrosion inhibition efficiency while lowering costs. Using a combination of inhibitors is a potential strategy for increasing corrosion inhibition efficiency at a lower cost. Several publications have investigated the synergistic inhibitory impact of inhibitor combinations. Fouda et al.^5^ studied the synergistic effect of iodide ions and aliphatic amines on corrosion inhibition of C-steel in sulfuric acid. They discovered that iodide ions boosted the adsorption of aliphatic amines on the metal surface, resulting in an increase in aliphatic amine inhibition efficiency^6^.

Tang et al.^7^ investigated that combining 8-hydroxyquinoline with Cl^−^ ions resulted in a synergistic inhibitory effect against cold-rolled steel corrosion in 0.5 M sulfuric acid. This results in increased inhibitory efficiency. Li et al.^8^ investigated the inhibitory effect of non-ionic surfactants and bromide ions on the corrosion of cold-rolled steel in sulfuric acid. They concluded that the interaction of non-ionic surfactant and bromide ion played a major role in corrosion inhibition. Villamil et al.^9^ observed that sodium dodecylsulphate and benzotriazole had a synergistic effect on copper corrosion inhibition in sulfuric acid. Marczewska et al.^10^ discovered that imidazoles, benzothiazoles, and mercaptobenzothiazoles are substantially effective corrosion inhibitors against pitting on steel samples in aqueous solutions to identify specific azole and thiazole compounds. They stated that these organic compounds are capable of building self-assembled monolayers, which increase inhibition and confer a very stable protective layer, due to the spontaneous self-assembly process. Calmon et al.^11^ patented mixtures against pitting and galvanic corrosion of copper and iron surfaces, consisting of sodium phosphates (Na_2_HPO_4_) or polyphosphates (Na_2_P_4_O_13_) that work synergistically with sodium mercaptobenzothiazole in aqueous solutions, emphasizing the direct relationship between inhibition efficiency and inhibitor concentration. Kartsonakis et al.^12^ reported the synergistic effect of the corrosion inhibition behavior of MBT and Na_2_HPO_4_ in a 1:1 molar ratio at various concentrations. The research found that the admixtures worked efficiently, with inhibition efficacy exceeding 90%^12^.

Ramji Karpagavalli et al.^13^ studied the synergistic corrosion inhibition of brass in 0.2 M NaCl solution by 2-mercaptobenzothiazole (MBT) and Tween-80 utilizing polarization, XPS and ICP studies. The investigation revealed that the combination inhibitor system exhibited a considerable improvement in inhibition efficiency up to 94% due to synergistic adsorption on the brass surface and suppression of dezincification. TSN Sankara Narayanan et al.^14^ studied the efficiency of MBT against dezincification corrosion of brass in the presence of sulfide polluted 3.5% NaCl media by electrochemical and accelerated leaching methods. Their results demonstrated that sulfide ions greatly inhibited the protective effect of MBT, due to the production of Cu_2_S films, which replaced the Cu-MBT complex layer. The synergistic effect of tolyltriazole (TTA) and nanoparticles as a corrosion inhibitor for brass alloy in a seawater media was examined by AbdElRhiem et al.^15^. An outstanding sample with remarkable corrosion resistance and a 99.03% efficiency rate in postponing the reaction to environmental stimuli is produced by the addition of SiO_2_. TiO_2_, on the other hand, has a moderate efficiency rate of 98.1%.

Gowrani et al.^16^ investigated the inhibitory behavior of 5-methyl benzotriazole (MBTA) for brass in 3% NaCl solution. Electrochemical and weight-loss tests showed that MBTA was a mixed-type inhibitor with Langmuir adsorption behavior and inhibition efficacy of up to 71%. Feng Wang et al.^17^ studied the influence of Al_2_O₃ nanoparticles on the corrosion prevention effectiveness of benzotriazole for brass. They found that raising the nanoparticle concentration reduced the BTA adsorption and lowered the inhibition efficacy due to interference with the creation of the protective coating. Alessia Artesani et al.^18^ examined the latest developments of protective coatings for cultural heritage applications, highlighting the contribution of nanoparticles, hybrid coatings and corrosion inhibitors to the improvement of long-term corrosion resistance and coating durability. Yu I Kuznetsov^19^ discussed the corrosion inhibition properties of benzotriazole derivatives for aluminum alloys and emphasized the role of adsorption and blockage of Cu-rich intermetallic sites in the corrosion prevention mechanisms. The suppression of brass in neutral chloride conditions by mercapto-triazole derivatives was studied by Ivan Arkhipushkin et al.^20^. XPS research verified the creation of a thin protective Cu/Zn-organic complex layer, which enhanced corrosion resistance substantially. Sarah B Ulaeto et al.^21^ examined the multifunctional role of nano-dispersoids in smart polymer nanocomposite coatings and demonstrated that nanoparticles enhance barrier characteristics, coating durability and corrosion protection efficacy.

Nanoparticles (e.g., TiO_2_, Al_*2*_O_*3*_) have been investigated individually for brass corrosion prevention, and are frequently dispersed in nanofluids with surfactants such as SDBS. These nanoparticles influence corrosion behavior in diverse ways based on their charge and interaction with surfactants and the brass surface^22,23^. The synergistic effect of BTA and nano Sb_2_O₃ on corrosion inhibition has been reported^24^. When exposed to a corrosive environment containing 5% NaCl, this combination increases barrier characteristics while also passivating the copper surface. The synergistic effect of Sb_2_O₃ and BTA was confirmed through open circuit potential (OCP), potentiodynamic polarisation, surface analyses using SEM-EDX and XPS, and electrochemical impedance spectroscopy (EIS). Results showed significantly reduced corrosion current densities and enhanced protective barrier formation. These findings show that the combination of organic molecules and inorganic nanoparticles increases the chemical stability of the copper surface while also improving its physical barrier properties. Combining BTA and Sb_2_O₃ nanoparticles at lower concentrations enhances copper-based materials’ corrosion resistance by approximately 99.96%^24^.

To further confirm the experimental results and also to get further insights into the corrosion inhibition mechanism at the molecular level, Density Functional Theory (DFT) simulations were done. DFT is a robust quantum mechanical tool for probing electronic structure, adsorption behavior and reactivity of corrosion inhibitors by means of important parameters such as frontier molecular orbitals (HOMO and LUMO), energy gap and global reactivity descriptors. In the present work, DFT calculations have been used to connect the electrical properties of 2-MBT and its nanoparticle-modified systems with the corrosion inhibition efficiency. This method has been highlighted by G. Gece, where Gaussian-based DFT calculations were used to predict the efficacy of inhibitors by means of electronic properties such as HOMO and LUMO energies^25^. Torres et al. obtained similar results by applying Gaussian DFT simulations to thiourea derivatives and showed that the high efficacy of corrosion inhibition is closely related to their electronic structure^26–29^.

However, there remains a lack of multidisciplinary studies combining 2-mercaptobenzothiazole (MBT) inhibitors and nanoparticles to investigate potential synergistic or negative effects on brass corrosion prevention. The interaction between MBT molecules and nanoparticles (TiO_2_ / SiO_2_) on brass surfaces is not generally understood and has not been documented till now. Addressing this gap could lead to more effective, robust, and versatile corrosion protection techniques for brass alloys.

Therefore, this research aims to explore the combined use of 2-mercaptobenzothiazole (MBT) inhibitors and 10 ppm TiO_2_ / SiO_2_ nanoparticles for brass corrosion protection in seawater medium. The corrosive electrolyte (3.5% NaCl) was applied to the samples at (2-MBT) inhibitor concentrations varying from 0 to 20 ppm. Electrochemical techniques such as OCP, EIS, and Tafel were used for corrosion evaluation. This study highlights the importance of the combination of adding nanoparticles and inhibitors (2 MBT) to achieve corrosion in aggressive media, showing excellent resistance and superior properties to the atmosphere, and shifting the potential in a positive direction. Moreover, DFT simulations were conducted to elucidate the inhibition mechanism at the molecular level and to correlate the corrosion inhibition efficiency of MBT and nanoparticle-modified systems with their electronic properties.

The originality of this work is to highlight that the selection of the obtained results shows that the semiconducting TiO_2_ nanoparticles exhibit a synergistic electrochemical inhibition mechanism with MBT molecules, leading to enhanced adsorption stability and formation of a compact protective film, while the insulating SiO_2_ nanoparticles contribute mainly by passive diffusion blocking. Moreover, the DFT analysis rationalizes the inhibitory performance of the studied systems by defining the function of electronic structure, charge distribution and adsorption propensity in the experimental data. This distinction provides useful insight for the design of advanced hybrid corrosion inhibitor systems, where the electronic properties of nanomaterials control the film compactness, the interfacial electrochemical stability, and the long-term corrosion resistance in corrosive environments.

## Experimental procedures

### Alloy chemical composition

Alpha and beta phases make up the two-phase structure of brasses that contain 35–45% zinc. X-ray photoelectron spectroscopy (XPS) was used to ascertain the brass alloy’s composition, as shown in Table 1.

**Table 1:** Lists the brass’s chemical makeup by weight% (wt%).

Sample	Cu	Zn	Pb	Si	Al
Brass alloy	59.10	39.8	0.8	0.04	Bal.

### Test solution

**Fig. 1:**
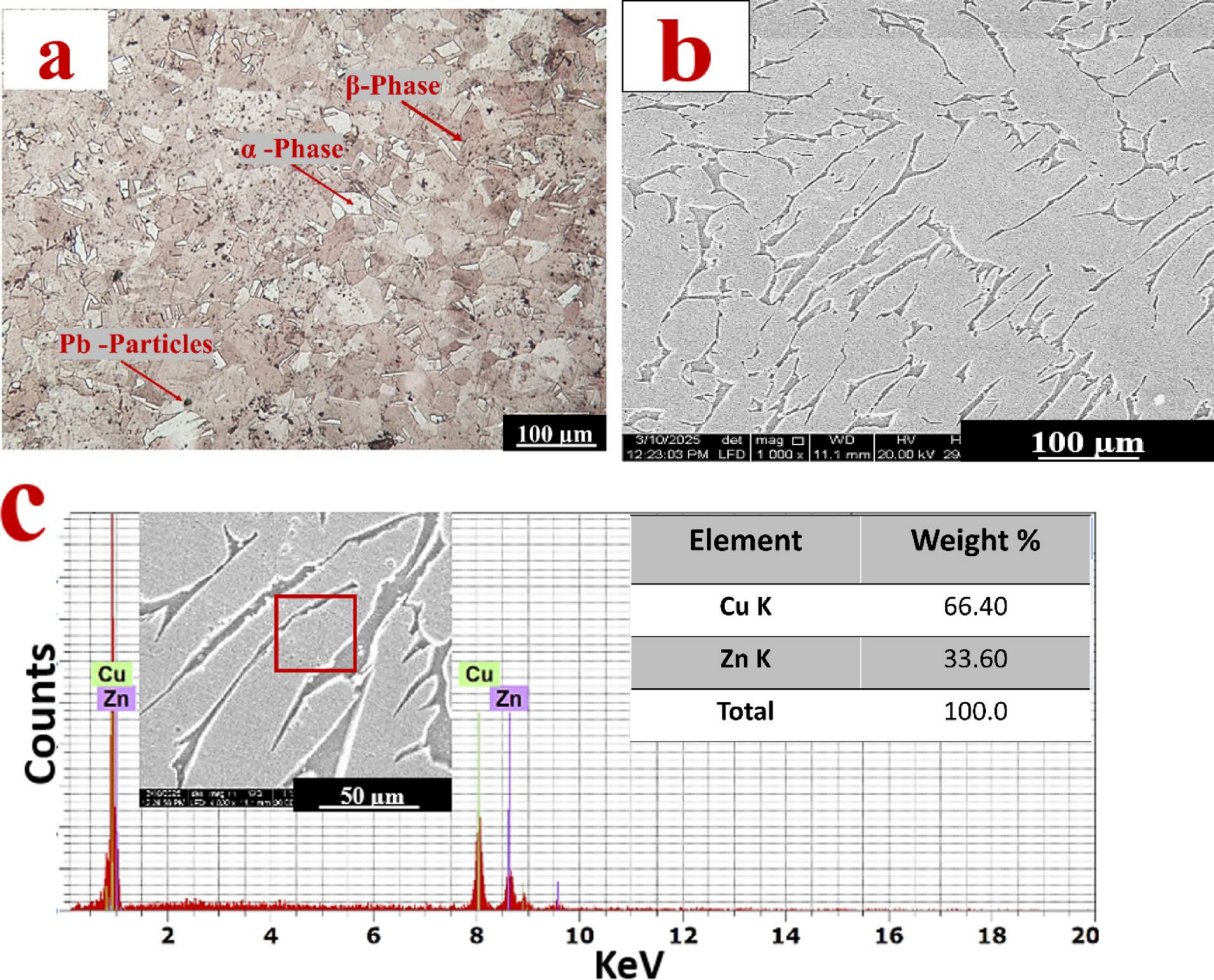
(**a**) OM, (**b**) SEM microstructure images, and (**c**) EdX analysis of brass alloy.

2-mercaptobenzothiazole (MBT), C_7_H_5_NS_2,_ is an essential type of thiazole rubber vulcanization accelerator. Simultaneously, it serves as a key auxiliary agent for oxidation and corrosion resistance, particularly in copper alloys^12^. It is very soluble in acetone. Also soluble in ethanol. Slightly soluble in benzene. The molecular structure of MBT is seen in Fig. 1. 2-MBT (99%) was obtained from Alfa Co. and utilized without additional purification. All solutions were prepared using 2-MBT inhibitor concentrations ranging from 0 to 20 ppm and dissolved in 100 mL of 99.7% ethanol before being added to a corrosion medium containing 3.5% NaCl. Finally, 10 mg of SiO_2_ and TiO_2_ nanoparticles will be exposed to the ideal condition (10 ppm) individually.

### A scanning electron microscopy (SEM)

The purpose of the study was to evaluate surface models and find structures. The Bruker AXS-Flash Detector 410-M in Germany is connected to the FEI Inspect S 50-Netherlands Energy Dispersive X-Ray Spectroscopy EDS.

### Transmission electron microscopy (TEM)

Images of nanostructured SiO_2_ and TiO_2_ powders were obtained using a JEOL JEM-2100 model, revealing that the additional components are nanoparticles. Panalytical X’pert BRO XRD has been used to identify the internal structure of the nanoparticles.

### LECO LX 31 optical microscope (OM)

LECO LX 31 Optical Microscope (OM) with 500 magnification.

### Electrochemical Investigations

#### Preparation of the brass samples

Brass sheets were purchased. Samples with dimensions (1 × 3 × 0.2 cm^3^). Each sample was numbered for ease of identification. The samples were polished first, then cleaned with distilled water and dried.

#### Corrosion test

To assess corrosion resistance in a 3.5% NaCl solution at room temperature, three electrodes were employed in an electrochemical testing station (Origafex-OGF01A-Origalys, France). The Pt sheet served as the auxiliary electrode, while the reference electrode was Hg/HgO. (OCP). Electrochemical impedance spectroscopy (EIS) measurements were conducted at the stabilized open circuit potential (OCP) to evaluate the electrochemical behavior of the systems under investigation within the frequency range of 0.1 Hz to 100 kHz. The impedance measurements were then used to extract the electrochemical time constant (τ), which is directly related to the charge transfer kinetics and the interfacial relaxation processes occurring at the metal/electrolyte interface. Tafel was utilized to evaluate the corrosion process for 30 min with a scanning rate of 0.002 V/s, with a possible range of − 0.5 to 0.5 V (vs. OCP).

### Statistical analysis

All electrochemical measurements were performed in triplicate under the same experimental conditions to confirm reproducibility. The acquired data were analyzed using Microsoft Excel and Origin Pro 2023 (Origafex-OGF01A-Origalys, France). We calculated mean values and standard deviations (mean ± SD) to assess the consistency and dependability of the findings. Graphical representations and data fitting were executed using Origin Pro software.

### Quantum chemical calculations

Density Functional Theory (DFT) computations were performed using B3LYP functional with LANL2DZ basis set as implemented in the Gaussian 09 W software package^30^. The B3LYP functional is a combination of the Lee–Yang–Parr correlation functional and Becke’s three-parameter hybrid exchange functional involving local, non-local and Hartree–Fock exchange contributions^31–33^. All molecular structures of MBT and its nanoparticle-modified systems were thoroughly optimized without any symmetry limitations. Vibrational frequency analysis was carried out to confirm the optimized geometries to rule out the presence of imaginary frequencies. The molecular structures and orbitals were visualized with Gauss View 6.0. The frontier molecular orbitals, including the highest occupied molecular orbital (HOMO) and the lowest unoccupied molecular orbital (LUMO), were obtained. Furthermore, certain important quantum chemical parameters such as energy gap (ΔE), dipole moment (µ) and Mulliken atomic charges were determined. The global hardness (η), global softness (s) and absolute electronegativity (χ) were also calculated. Calculations were performed in a 3.5% sodium chloride (NaCl) aqueous solution.

**Scheme 1:**
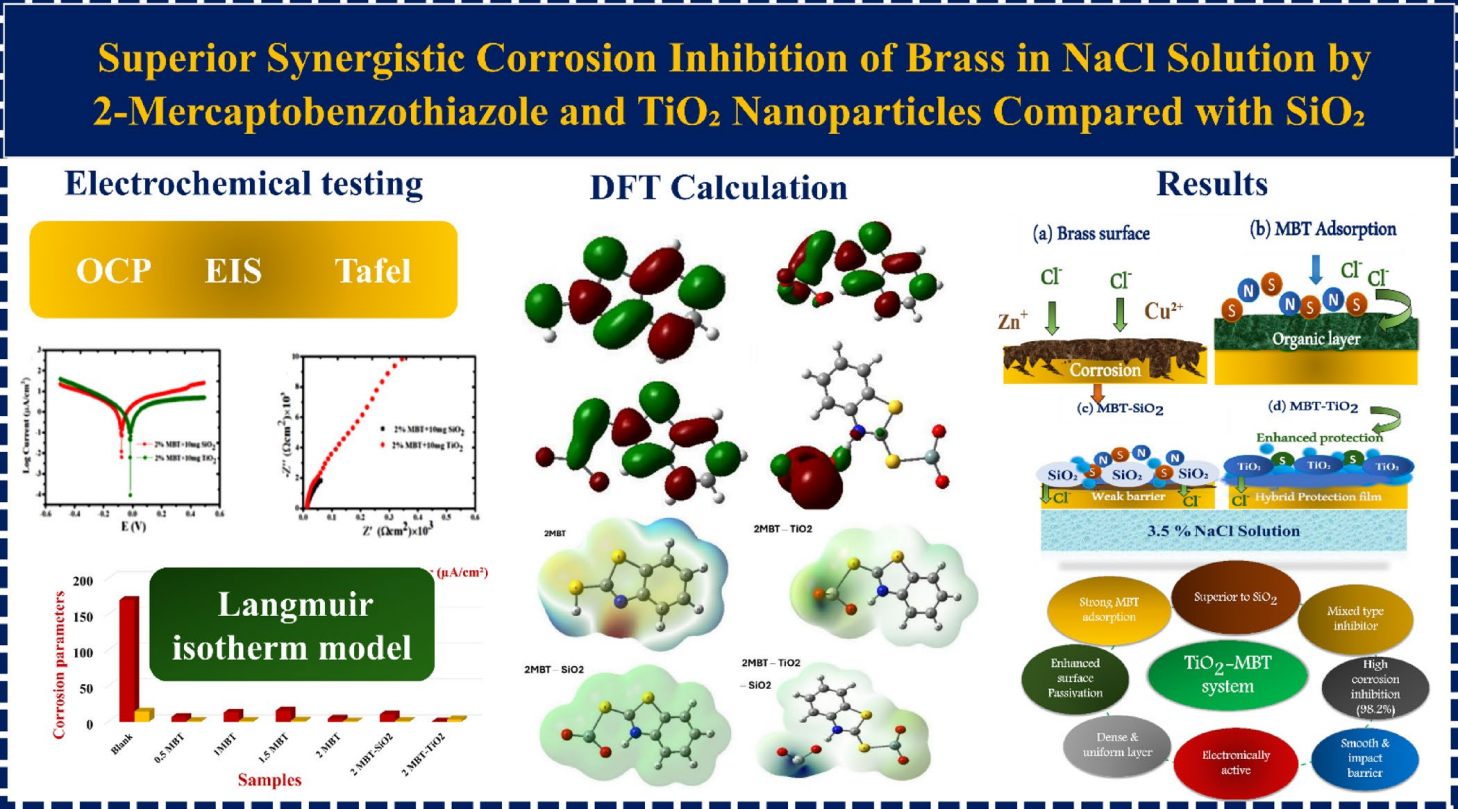
The synergistic effect of 2-mercaptobenzothiazole (MBT) and nanoparticles as a corrosion inhibitor for brass alloy in sodium chloride solution (Schematic illustration prepared using Microsoft PowerPoint software).

The synergistic effect of 2-mercaptobenzothiazole (MBT) and nanoparticles as a corrosion inhibitor for brass alloy in sodium chloride solution is illustrated in Scheme 1.

## Results and discussions

### Morphology of brass alloy and nanoparticles

Brasses are copper-zinc alloys in which the primary alloying element is zinc, which ranges from 5 to 40%. In a face-centered cubic (fcc) copper matrix, zinc concentrations of up to 35% can dissolve and form a single solid solution (α brass). A β second phase with a body-centered cubic (bcc) lattice structure is formed when more zinc is added. Compared to α brasses, duplex α + β brasses are more affordable, simpler to produce, and have better mechanical strength. Yet, as Fig. 1a illustrates, the zinc-rich β phase is more prone to dezincification corrosion, which results in a porous layer of copper, weakening the component and causing brittle failures^34^. Furthermore, lead additions improve alloy machinability by up to 3%, but above that point, lead has detrimental impacts on casting properties, including hot ripping and shrinkage^35^. Cheap commercial brasses, on the other hand, usually contain more than this amount. In copper-zinc alloys, lead is insoluble and precipitates during solidification, forming globules inside the matrix and along the borders of grains. It may improve corrosion resistance or have no impact, depending on the alloy composition and environment^35^.

**Fig. 2:**
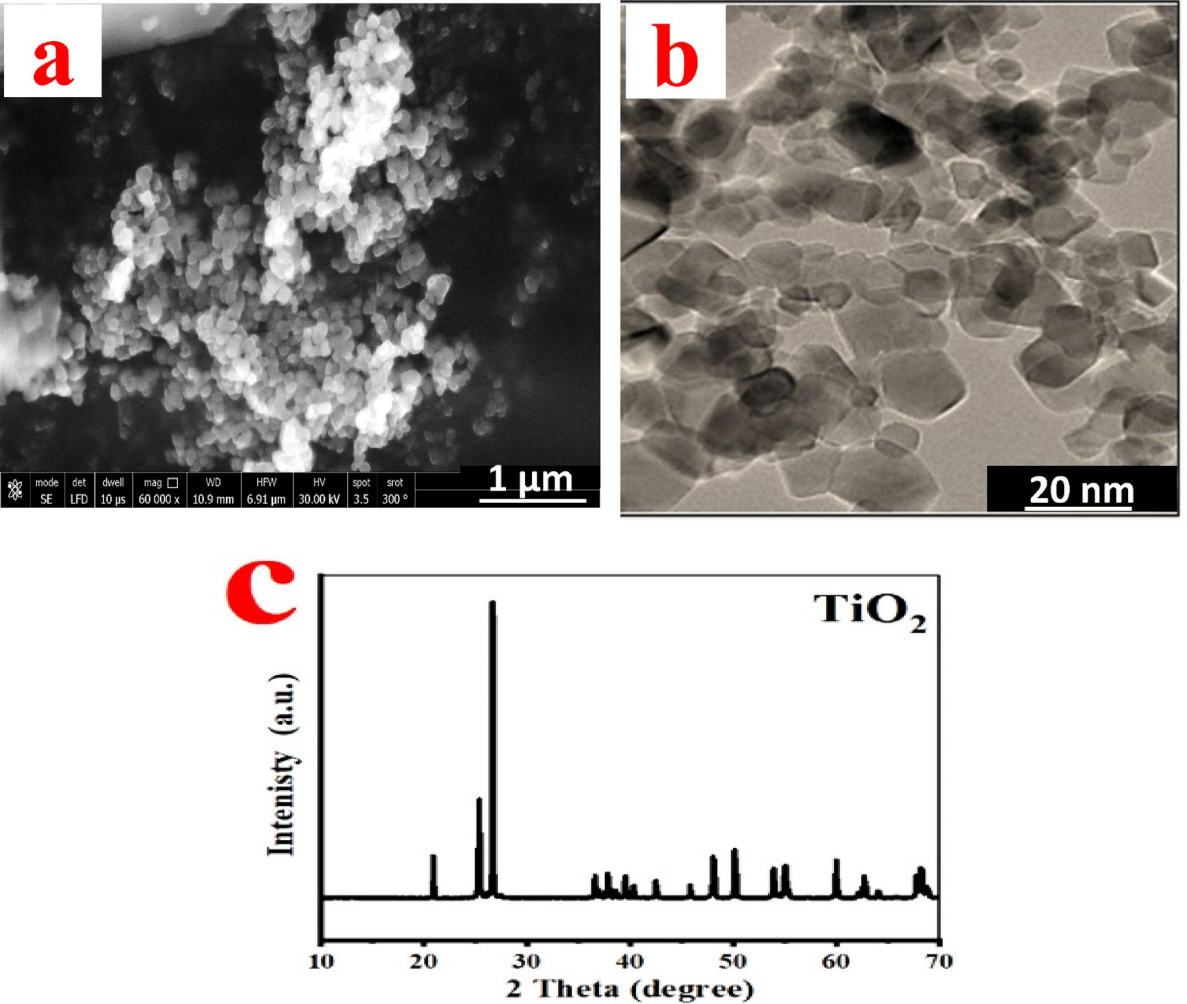
(**a**) SEM, (**b**) TEM images, and (**c**) XRD pattern of TiO_2_ NPs.

**Fig. 3:**
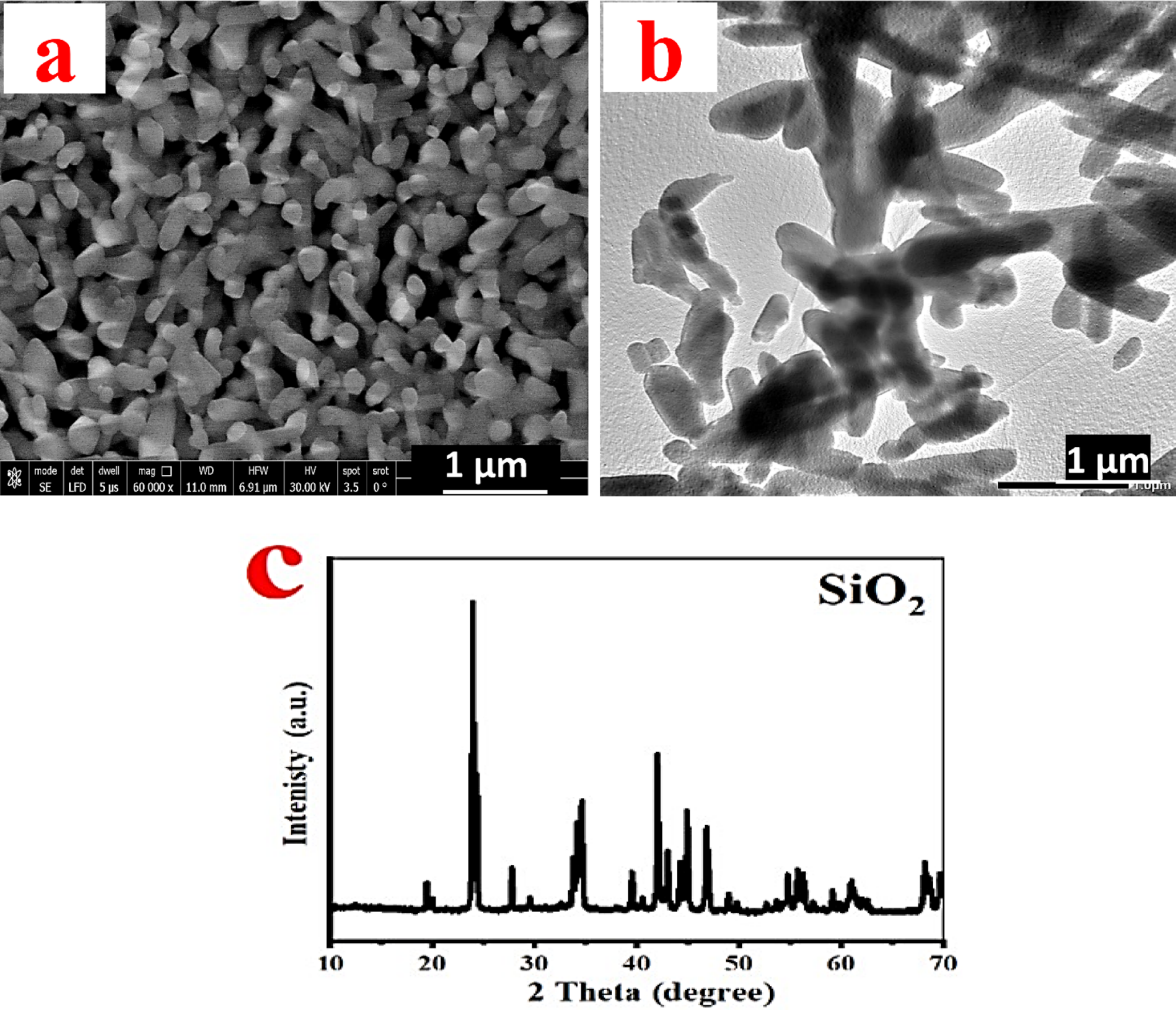
(**a**) SEM, (**b**) TEM images, and (**c**) XRD pattern of SiO_2_ NPs.

SEM and TEM were used to analyze the crystal structure and microstructure of nanostructures (NSs). Figures 2 and 3a and b show the structures of SiO_2_ and TiO_2_, which appear as nanorods and nanoparticles, respectively. Also, demonstrate that the NSs at the nanoscale range from 20 to 80 nm. Figures 2 and 3c depict the XRD patterns of SiO_2_ and TiO_2_, which show a hexagonal SiO_2_ phase and a tetragonal TiO_2_ phase. The XRD patterns reveal strong peaks, indicating that the nanostructured powders are extremely crystalline^36^.

### Electrochemistry measurements

#### Potentiodynamic polarization analysis

**Fig. 4:**
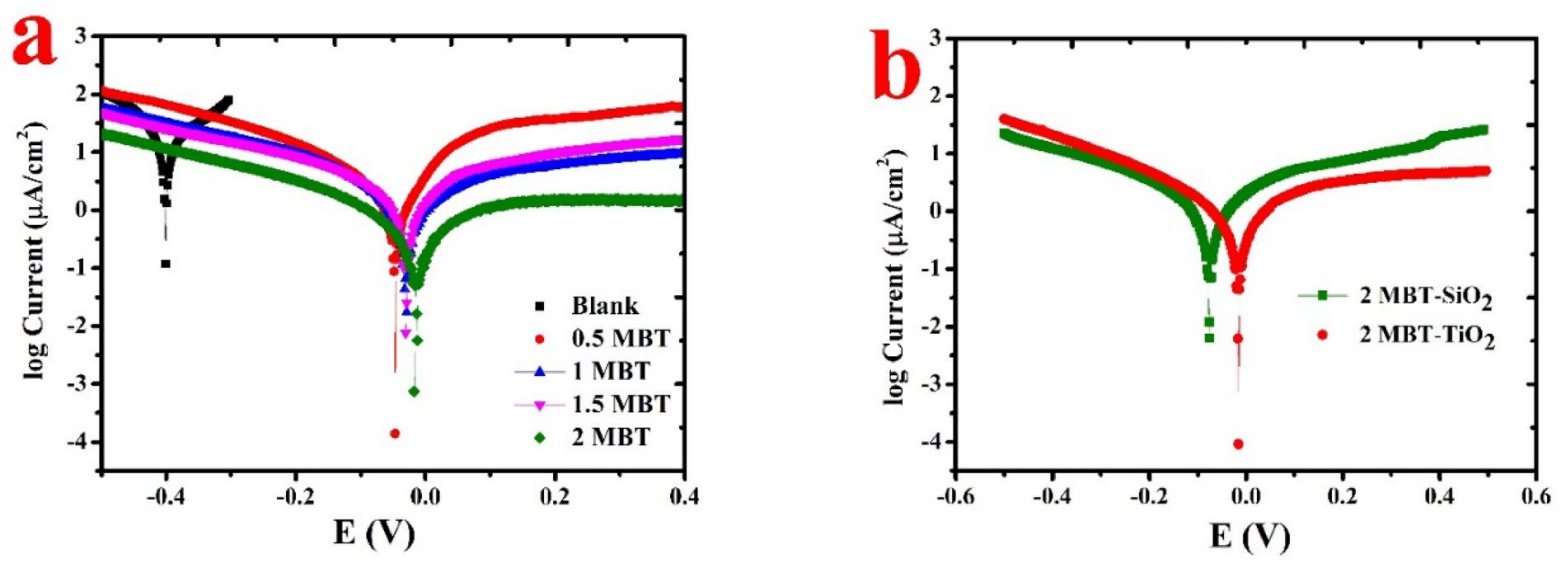
Potentiodynamic polarization curves (**a**) blank, MBT at different concentrations, and (**b**) 2 MBT with the addition of SiO_2_ and TiO_2_ nanoparticles in 3.5% NaCl solution.

**Fig. 5:**
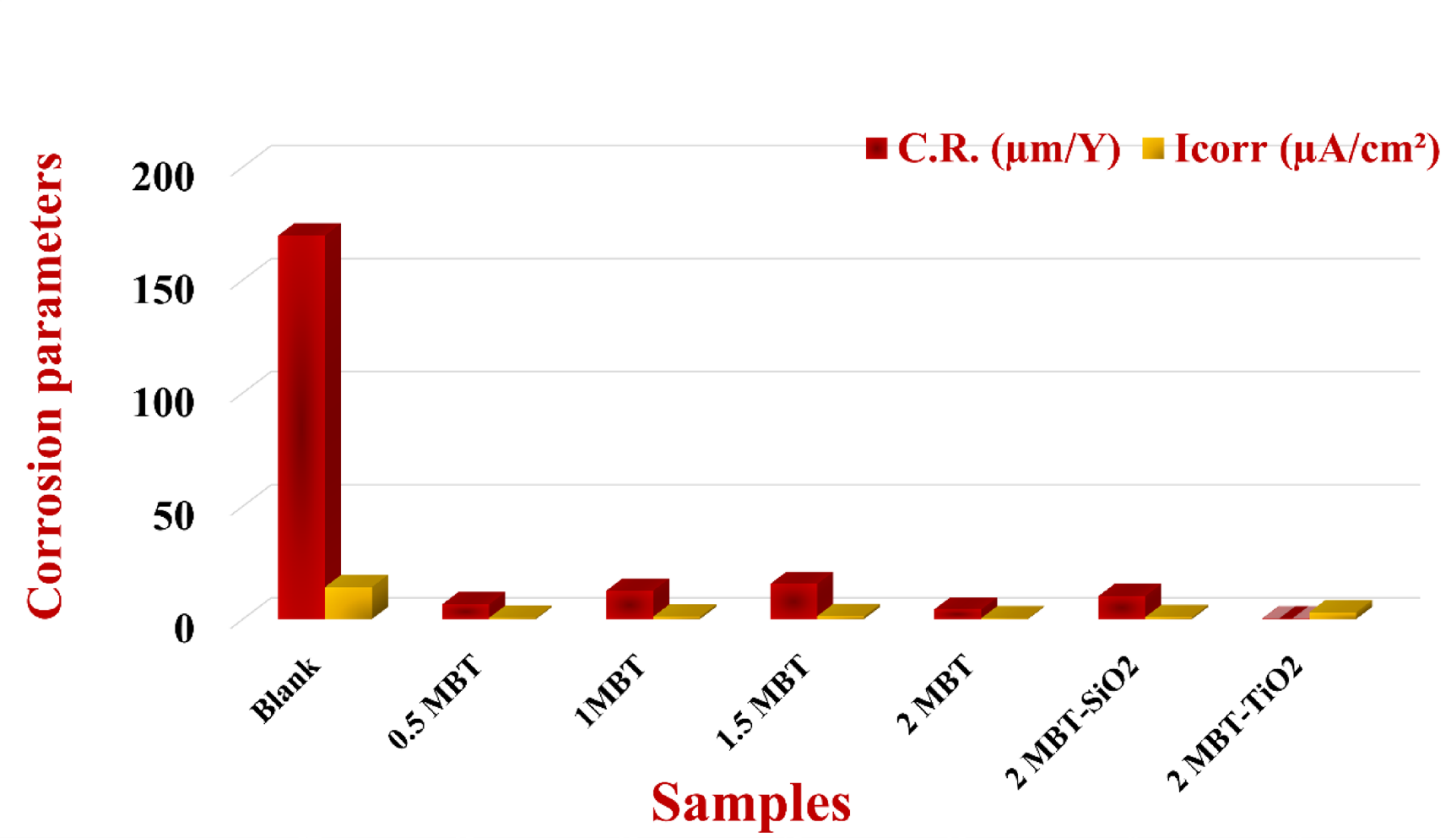
Variation of corrosion rate and corrosion current density with the concentration of the studied inhibitors for brass alloy in 3.5% NaCl.

Figure 4 depicts the Tafel curves for brass in sodium chloride solution, both with and without MBT, as well as nano addition at various concentrations. Table 2 displays the electrochemical characteristics, such as the cathodic and anodic corrosion potential (E_corr_.), that were determined from the polarization curves. Tafel slopes (β_a_ and β_c_), corrosion current density (I_corr_.), and inhibition efficiency (η). Inhibitors led to a significant decrease in I_corr_. values from 14.1898 µA/cm^2^ to 0.3281 µA/cm^2^ in Fig. 5; Table 2, revealing a further reduction at higher inhibitor levels.

Figure 11 shows that η as the inhibitor level grew, consequently the maximum value at 2 MBT (97.4%) and incorporating 2MBT with 10 mg TiO_2_ (98.2%), indicating a bigger number of molecules for the inhibitor surrounding the metal’s surface^37^. In Fig. 4; Table 2, the potential corrosion values were compatible, with a fluctuation in E_corr_ of ~ 86% between the 0 and the inhibitor values, indicating a mixed-type inhibitor. Furthermore, this is predicted according to the attachment of inhibitor molecules, which form an obstacle layer and the nanoparticles precipitate, blocking the sites that are active and pores on the metal substrate^37,38^. Because of the presence of surfactants in their structures, MBT at all concentrations and with nano additions was superior with respect to preventing corrosion compared to the blank, where this combination could contribute to decreasing the alloy dezincification. As mentioned before, organic inhibitors function by adsorbing on the solution/metal interface, which provides information on the inhibitor’s interaction with the surface^37^. The polarization curves showed that these inhibitors bind to the metal surface due to the presence of N and S atoms in the thiazole ring^12^, all of which supply strong adsorption centers and improve the protective layer thickness. Although this yellow inhibitor will oxidize and deplete, especially in an open corrosive medium. Nanoparticle addition can stop this problem.

MBT, as a corrosion inhibitor, creates a strong electrochemical layer on brass surfaces, offering excellent resistance to corrosive media such as NaCl solutions. This protective film on metal surfaces from MBT acts as an anodic inhibitor, delaying anodic processes and preventing metal breakdown^12^. When MBT accumulates in a corrosive medium, the corrosion potential moves to higher anodic values, reducing corrosion current densities^37^. Inhibitor 2-mercaptobenzothiazole (MBT) links with metal ions via N and S atoms in MBT and subsequently adsorbs on the metal’s surface, inhibiting corrosion of copper alloys^39^.

Thus, the MBT concentration rises, a protective layer (Cu (II)) forms, lowering corrosion rates^39^ and shifting the samples to a more positive potential, as illustrated in Fig. 4a. In cases in which the addition of nanoparticles reduces the surface uniformity of the brass where MBT is encapsulated, insignificant amounts of suppressive nanoparticles are introduced. This enables regulated release in response to environmental stimuli, hence extending the protective advantages, as shown in Fig. 4b^38,40^. Adding 10 mg nanoparticles to the optimum concentration (2 MBT) of brass improved corrosion resistance, particularly with TiO_2_. The efficacy changes with nanoparticle additions.

The addition of nanoparticles in the corrosive medium results in a higher corrosion inhibition performance of MBT, as shown in Figs. 4b, 7b, 8b and 9b, and 10b, especially the SiO_2_ addition. This occurs because SiO_2_ nanoparticles can provide full surface coverage by filling pores and imperfections^41^. According to studies, optimal nanoparticle concentrations are essential for optimizing inhibitory efficiency, as excessive concentrations can interfere with adsorption processes and decrease the stability of protective films, especially TiO_2_^42^. To modify the surface homogeneity of the brass where MBT is encapsulated, small to moderate ratios of suppressive nanoparticles are added. This allows for controlled release in response to environmental stimuli, prolonging the protective benefits^43^.

### Anodic and cathodic kinetics and inhibition mechanism

Figure 4a, b and Table 2 indicate that the corrosion inhibition depends on the modification of the anodic and cathodic kinetics by adsorption and film formation.

The blank sample has identical Tafel slopes (βₐ ≈ β_c_ = 142.8 mV) and a high I_corr_, indicating that both processes proceed freely under activation control. βₐ (anodic Tafel slope) is the sensitivity of metal dissolution (M → Mⁿ⁺ + ne⁻) to potential, and its moderate value indicates that the oxidation process is quick and not substantially impeded. β_c_ (cathodic Tafel slope) is the indicator of the kinetics of oxygen reduction. The similar value shows the free electron transfer on the surface^24,44^.

The introduction of MBT (0.5) leads to a considerable reduction of both β_a_ and β_c_. The surface is partially covered, and as a result, the sensitivity of the electrochemical responses to the potential variations decreases. The fall in β_a_ means that anodic dissolution sites are hindered, and the decrease in β_c_ demonstrates that oxygen reduction sites are also impeded^45^. This behavior is related to the physisorption, where MBT creates the initial adsorbed layer, which functions largely as an insulating organic barrier, lowering the active conducting metallic surface and decreasing the charge transfer^17,24^.

For high MBT concentrations (1–1.5), β_a_ increases (162–205 mV) and β_c_ becomes more negative. With a higher value of βₐ, the anodic dissolution now needs a bigger overpotential, suggesting a stronger surface binding and a higher energy barrier because of the creation of the film. The more negative the β_c_, the stronger the suppression of the cathodic process since it is more difficult to transport electrons to the oxygen^46^. This stage is attributed to chemisorption in which MBT (via S and N atoms) establishes coordination bonds with the metal, leading to a more stable and compact film that acts as a strong insulating-blocking layer with limited paths for charge transport. Local defects in the film are shown by very small changes in I_corr_.

At the optimum (2 MBT), βₐ is at its maximum (217.7 mV), whereas β_c_ is still significantly negative. I_corr_ is at its minimum. This indicates a very compact chemisorbed layer that greatly enhances the activation barrier for both anodic and cathodic processes^26^.

For the nanoparticle modified systems, in the SiO_2_ system, β_c_ is quite negative, i.e., the cathodic reaction is strongly inhibited. SiO_2_ is a very insulating oxide and improves the surface blocking specifically for sites of electron transport. However, the porous structure permits some penetration of the electrolyte, which is responsible for the significantly greater I_corr_. SiO_2_ largely enhances insulation and site blocking but not film compactness.

Meanwhile, the TiO_2_ system exhibited the lowest I_corr_ and balanced β_a_ and β_c_ values. TiO_2_ is a semiconducting oxide that leads to a more homogeneous, compact and electronically stable composite layer. It decreases the defect density and enhances the film cohesiveness, so it more effectively suppresses both anodic and cathodic processes concurrently.

In summary, β_a_ is a measure of the resistance to the oxidation of metal (anodic dissolution), and βc is a measure of the resistance to electron-consuming reduction processes^44^. Their fluctuation illustrates the transition from weak physisorption (insulating but loose covering) to strong chemisorption (compact blocking covering). Nanoparticles modulate the balance between insulating behavior (SiO_2_) and compact semiconducting protection (TiO_2_), as confirmed in Fig. 12.

The corrosion parameters and the C.R. were determined using Tafel extrapolation measurements, as shown in Fig. 4a, b. Equation (1) is used to determine the C.R. [ 34, 38].

1 $${\mathrm{C}}.{\mathrm{R}}.{\text{ }}=\frac{{0.0032{\mathrm{~}} \times {\mathrm{~Icorr}}.{\mathrm{~}} \times {\mathrm{~}}\left( {{\mathrm{M}}.{\mathrm{W}}.} \right)}}{{{\mathrm{n~}} \times {\mathrm{~d~}}}}$$

M.W. is the deteriorated material’s molecular weight (g mol^−1^), C.R. is the corrosion rate (mpy), and I_corr_. is the current density of corrosion (mA cm^−2^), n is the number of charge transfers throughout the corrosion process, and d is the density (g cm^− 3^)^34^. R_p_ increases with MBT content (89.99 kΩ cm²), while the sample without additions has the minimum R_p_ value of 1.08 kΩ cm², indicating corrosion resistance and shifting the samples to a more positive potential^40^.

Equation 2 used to calculate polarization resistance (R_p_) is^47^.

2 $${\mathrm{Rp~}}=\left( {~\frac{{{{{\Delta}E}}}}{{{{{\Delta}I}}}}} \right)~~~{{\mathrm{E}}_{{\mathrm{corr}}.}}=~~\left( {\frac{1}{{{{\mathrm{I}}_{{\mathrm{corr}}.}}}}} \right)~~~~{\mathrm{~}}\frac{{ - {\beta _c}+{\beta _a}}}{{2.3~\left( { - {\beta _c}+{\beta _a}} \right)}}$$

ΔE and ΔI indicate the polarization potential (V) and current density, respectively. E_corr_. is the self-corrosion potential (V), and β_a_ and β_c_ are the Tafel anode and cathode constants, respectively. The higher the R_p_ value, the better the corrosion resistance^34^, as shown in Table 2.

**Table 2:** Potentiodynamic polarization parameters for the brass alloy samples in 3.5% NaCl solution.

System	Conc. ppm		*R*_*p*_ kΩ cm²	β_a_ mV	β_c_ mV	E(i = 0) mV	I _corr_. _µA cm_^−2^	C.*R*. µmy^− 1^	θ		η %	Mean ± SD
Blank	–	1.08		142.8	142.8	− 117.4	14.189	169.6	–		–	0.04
0.5 MBT	5	16.77		55.5	− 57.9	− 47.1	0.547	6.5	0.961		96.1	0.003
1 MBT	10	29.49		205.4	− 153.0	− 29.4	1.050	12.5	0.926		92.6	0.005
1.5 MBT	15	23.71		162.2	− 179.8	− 29.9	1.301	15.5	0.908		90.8	0.002
2 MBT	20	89.99		217.7	− 165.6	− 14.2	0.359	4.3	0.974		97.4	0.003
2 MBT-SiO_2_	20 MBT 10 SiO_2_	38.95		188.0	− 192.1	− 75.9	0.849	10.1	0.940		94.0	0.007
2 MBT-TiO_2_	20 MBT 10 TiO_2_	56.99		116.7	− 110.3	− 16.5	0.328	3.0	0.982		98.2	0.002

Figure 5 shows the synergistic effect of 2-mercaptobenzothiazole (MBT) and nanoparticles as corrosion inhibitors for brass alloy in sodium chloride solution, verifying the Tafel plots and corrosion characteristics. The exhibited E_corr_, I_corr_, and C.R. values decrease as the concentration of MBT increases^37^, as shown in Table 2. As a corrosion inhibitor, the synergistic MBT and nanoparticles form a strong electrochemical protective layer on brass surfaces, providing superior protection against corrosive media such as NaCl solutions with nano addition, in addition to enhancing corrosion resistance for alloys^38^.

### Adsorption isotherm analysis

The adsorption behavior of 2-mercaptobenzothiazole (MBT) on the brass surface was investigated using adsorption isotherm models in order to better understand the inhibition mechanism.

The surface coverage (θ) was calculated from the inhibition efficiency (η%) obtained from potentiodynamic polarization measurements using Eq. (3)^27–29^:

3 $$\theta ~~=~~\frac{\eta }{{100}}$$

The obtained θ values were fitted to the Langmuir adsorption isotherm, expressed in Eq. (4)^29^:

4 $$\frac{{{C_{inh}}}}{\theta }=~\frac{1}{{{K_{ads}}}}+C{~_{inh}}$$

where C_inh_ is the inhibitor concentration, and K _ads_ is the adsorption equilibrium constant.

The concentration of MBT was expressed in ppm and used consistently throughout all adsorption isotherm calculations.

**Fig. 6:**
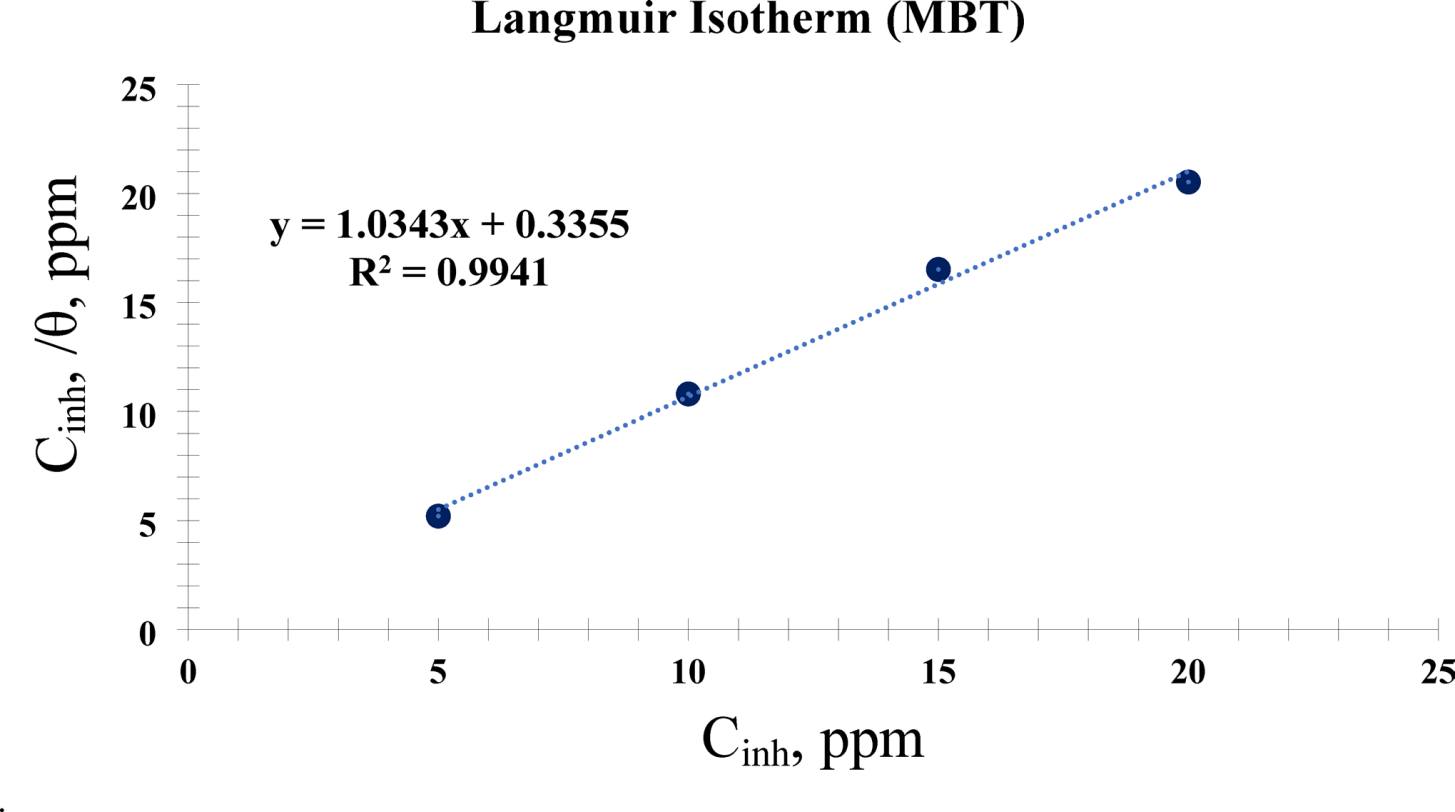
Langmuir adsorption isotherm plot (C_inh_/θ vs. C_inh_) for MBT adsorption on brass surface in 3.5% NaCl solution.

The linear plot of C_inh_/θ versus C_inh_ (Fig. 6) showed a good conformity with the Langmuir model, with a slope of ≈ 1.0343, slight deviation due to surface heterogeneity, and a high correlation coefficient (R^2^ = 0.9941), indicating that the adsorption process approximately follows the Langmuir behavior with the formation of a near-monolayer coverage on the brass surface^27–29^.

The adsorption equilibrium constant (K_ads_) was calculated from the intercept of the linear plot and was found to be 2.98 L mol⁻¹, reflecting strong adsorption of MBT molecules and the brass surface. Causing superior corrosion inhibition and stable film formation.

The standard free energy of adsorption (ΔG°_ads_) was calculated using the following Eq. (5)^27–29^:

5 $$\Delta {\mathrm{G}}^\circ _{{{\mathrm{ads}}}} = {\text{ }} - {\text{RT ln }}({\mathrm{55}}.{\text{5 K}}_{{{\mathrm{ads}}}} )$$

where R is the universal gas constant, T is the absolute temperature, and 55.5 represents the molar concentration of water in the solution^28^.

The computed value of ΔG°_ads_ (-12.66 kJ /mol) shows that the adsorption process is mostly physisorption. However, the considerable changes in Tafel slopes and the increase in inhibition efficiency at higher concentrations indicate the creation of a more stable and highly adherent layer, suggesting the role of chemisorption. Therefore, the adsorption mechanism can be expressed as mixed adsorption with physical and chemical interactions, as the Tafel test confirmed it.

The nanoparticles, which operate as modifiers of the MBT adsorption layer rather than as primary adsorbing species, were not included in the adsorption isotherm modeling.

#### Open circuit potential (OCP) behavior

**Fig. 7:**
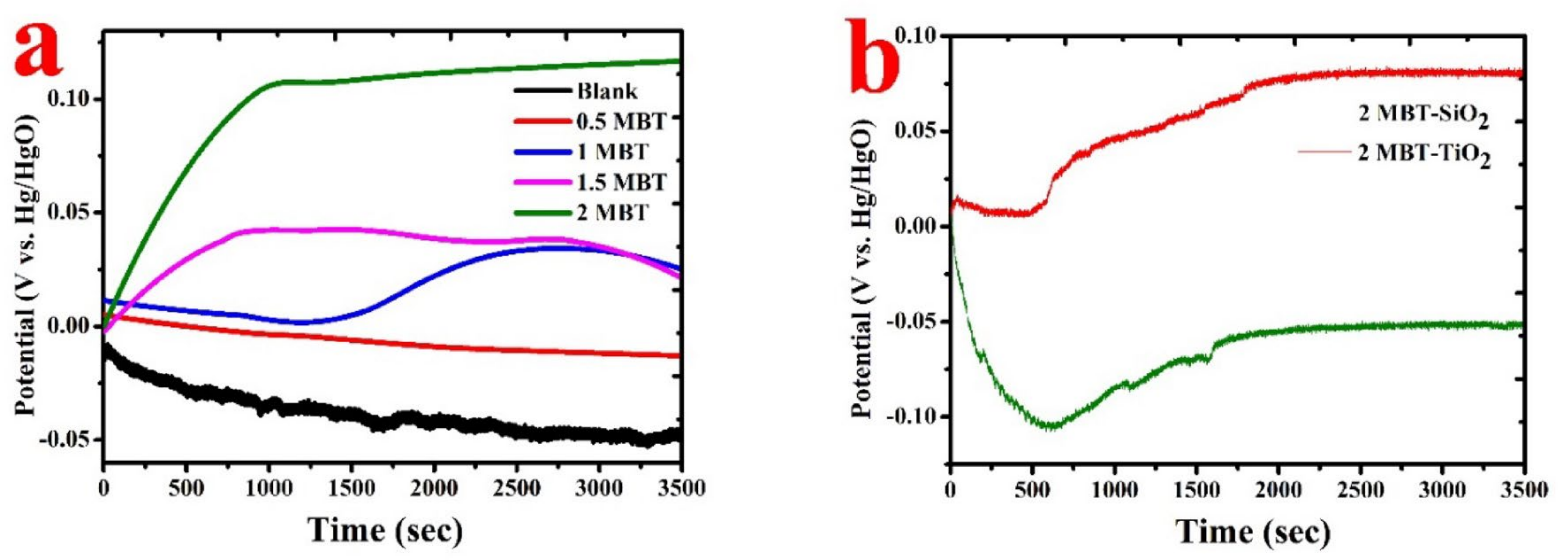
OCP curves (**a**) blank, MBT at different concentrations, and (**b**) 2MBT with nanoparticles SiO_2_ and TiO_2_ addition samples in 3.5% NaCl.

Figures 7a and b show the OCP curves for samples without additives, samples with different MBT concentrations, and samples with nanoparticle addition. The OCP values of the samples were recorded over 3500s. Compared to the blank sample, the OCP increased steadily as the concentrations of MBT and the value-added by adding 10 ppm of nano (SiO_2_, TiO_2_) increased. MBT was added to the blank sample at various doses, including 2%, which increased its potential from − 0.01367 to 0.0152 vs. Hg/HgO. The best sample’s optimum value was 10 ppm TiO_2_, resulting in a potential change from − 0.01367 to 0.00684 V vs. Hg/HgO.

Tafel parameters and plots are supported by OCP curves that show the effects of adding both 2MBT and nanoparticles, which cause the potential to shift towards positive values^40^, with nanoparticles modifying interaction with metal surfaces by increasing the surface area available for adsorption^38^. As a result, the corrosion resistance of brass alloys can be significantly improved by successfully incorporating nanoparticles and MBT inhibitor^37–39^.

#### Electrochemical impedance spectroscopy (EIS)

**Fig. 8:**
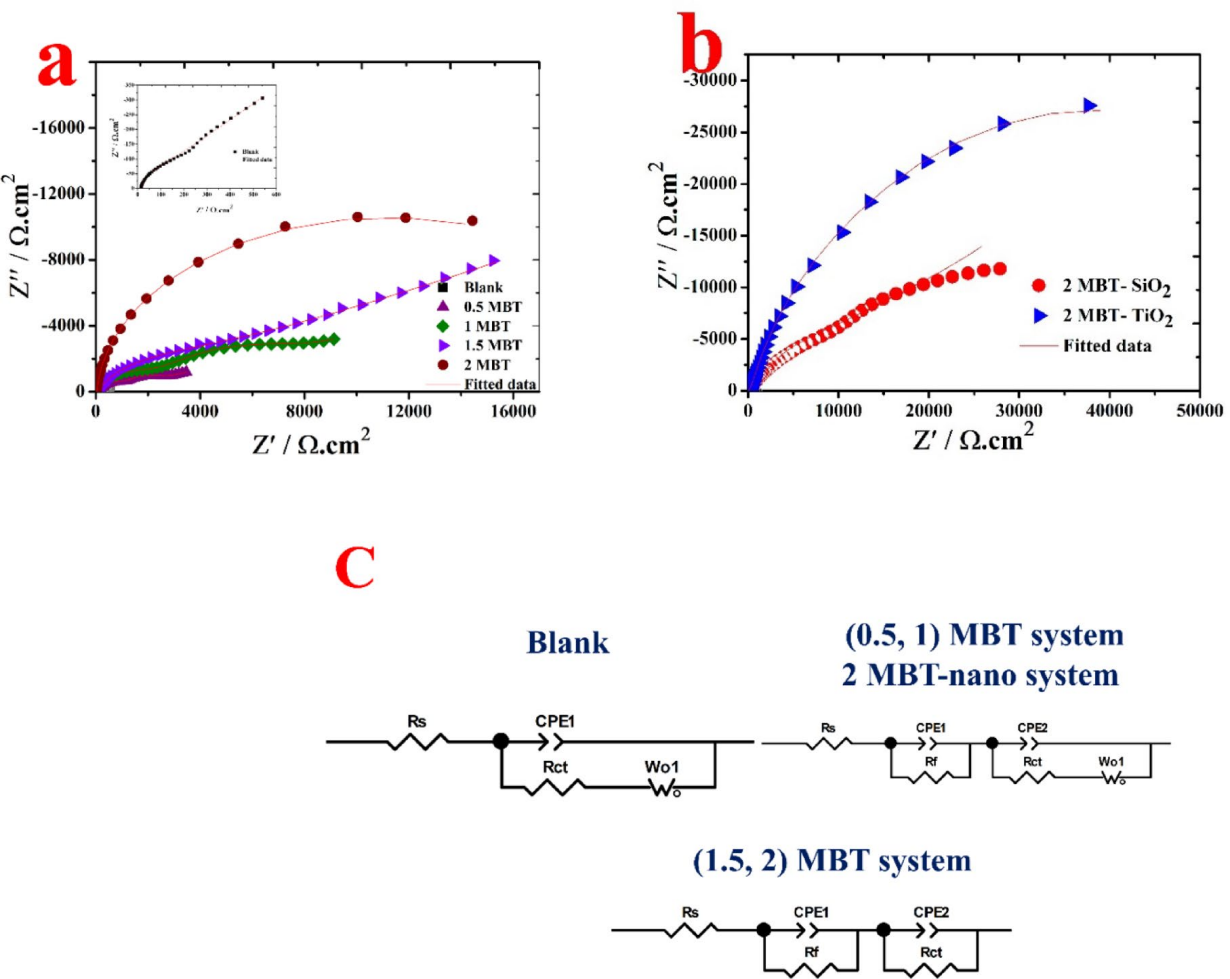
Nyquist impedance spectra for brass measured in 3.5% NaCl solution in the absence and presence of different concentrations of the studied inhibitors (**a**) MBT, (**b**) nanoparticles addition with 2MBT, and (**c**) equivalent circuits used to fit the impedance spectra.

The electrochemical impedance spectroscopy (EIS) in Fig. 8 results offered detailed information on the corrosion inhibition behavior of MBT, and MBT-nanoparticle hybrid systems for brass immersed in 3.5% NaCl solution^48^. All the systems (Fig. 8) showed depressed capacitive semicircles in the Nyquist plots, pointing to non-ideal capacitive behavior owing to surface heterogeneity^49^, adsorption processes and interfacial roughness. The rise in diameter of the semicircle after the addition of the inhibitor indicated the improvement in corrosion resistance due to the creation of protective adsorption layers on the brass surface^43^. The blank sample showed the minimum semicircle with only 690.4 Ω cm^2^ of charge transfer resistance (R_ct_), which proved the fast electrochemical corrosion in chloride media. The equivalent circuit of the blank system was composed of solution resistance (R_s_), charge transfer resistance (R_ct_), constant phase element (CPE_ct_) and Warburg diffusion element (Wo), which suggests that the corrosion process is mainly controlled by charge transfer and diffusion processes^50^.

The impedance response rose dramatically after the addition of the MBT inhibitor, which confirmed the adsorption of MBT molecules onto the brass surface and suppression of electrochemical reactions. The 0.5 MBT system demonstrated an increase in (R_ct_ = 200 Ω cm^2^) and the establishment of a film resistance R_f_ to 1042 Ω cm^2^ characteristic of the first generation of an adsorbed protective layer. The corrosion resistance further increased with the increase of the inhibitor concentration to 1 MBT^4,37^. R_f_ and R_ct_ were achieved at 1233 and 1133Ω cm^2^, respectively. This development proves higher surface coverage and the formation of a more continuous protective film^28^. The 1.5 MBT system showed an unexpected rise in R_f_ to 7974 Ω cm^2^ and R_ct_ to 2321 Ω cm^2^, indicating the production of a highly resistive interfacial layer, which may greatly inhibit electrochemical charge transfer reactions. The optimum performance of the pure MBT systems, however, was achieved for the 2 MBT formulation, where a high R_ct_ value (7658 Ω cm^2^) was coupled with the highest film resistance of the MBT-only systems (R_f_ =14168 Ω cm^2^). The high R_f_ value indicated that the layer of inhibitor was dense and sticky and was very resistant to the penetration of electrolyte^51^.

The equivalent electrochemical properties are listed in Table 3.

Since the double layer at the interface does not behave like an ideal capacitor, we used a constant phase element (CPE) in our circuits rather than pure double-layer capacitance to get a more accurate match. The CPE’s impedance function is represented by Eq. 6^52^.

6 $${{\mathrm{Z}}_{{\mathrm{CPE}}}}=\frac{1}{{Y~{{\left( {JW} \right)}^n}}}$$

where j is the imaginary unit, W is the angular frequency, n is the deviation parameter between 0 and 1, and Y is the admittance magnitude of the CPE constant. Z_CPE_ is comparable to a perfect Warburg impedance, resistance, and capacitor^53^.

The equivalent circuit for all inhibited systems was R_s_ in series with two-time constants: the high-frequency loop was attributed to R_f_ and CPE_f_, representing the protective inhibitor film, while the low-frequency loop was associated with R_ct_ and CPE_ct,_ corresponding to the electrochemical charge transfer process. The production of adsorption films upon the addition of the inhibitor was confirmed by the presence of two-time constants. The high frequency response was related to the dielectric characteristics and compactness of the inhibitor covering, whereas the low frequency response was related to the corrosion reactions at the metal/electrolyte interface^54^.

The constant phase element parameters proved the outcomes of impedance. The reduction of the CPE-T values following the addition of the inhibitor suggested the reduction of the local dielectric constant and/or the rise of the thickness of the electrical double-layer due to the displacement of water molecules by adsorbed MBT species^54^. Moreover, the CPE exponent values (n) approaching unity confirmed a comparatively capacitive behavior and better surface uniformity. The inhibited systems showed the more stable and less permeable interfacial films compared to the blank sample.

The addition of the nanoparticles strongly affected the electrochemical behavior of the inhibitor system^17,18,21,24^. Among all the studied systems, the 2 MBT–TiO_2_ formulation presented the best corrosion protection performance. The system exhibited a very high R_f_ value of 66,614 Ω cm² and a high R_ct_ of 4000 Ω cm², demonstrating the production of a very compact and robustly protective barrier layer^55^. The TiO_2_ system has maximum Warburg resistance (Wo-*R* = 82225 Ω cm^2^), i.e. strong inhibition of diffusion of chloride ions through the protective layer. Moreover, the relatively high values of the CPE exponent confirmed the increased surface homogeneity and more uniform interfacial properties. The performance of the TiO_2_-containing system is improved by the synergistic interaction of TiO_2_ nanoparticles with MBT molecules^24^. The TiO_2_ nanoparticles probably filled the pores and structural imperfections of the inhibitor layer, increased the compactness of the film, improved the adsorption stability and reduced the ionic transport channels across the layer^17,18^.

However, the corrosion resistance of the 2 MBT-SiO_2_ system was much lower than that of the 2 MBT system, even with the addition of the nanoparticles. The film resistance (R_f)_ value reduced to only 871.5 Ω cm^2^, and the R_ct_ was limited to 1120 Ω cm^2^. Although the SiO_2_-containing system displayed a relatively strong diffusion resistance, its electrochemical protection was still much lower than that of the TiO_2_-containing system. This behavior suggested that SiO_2_ nanoparticles largely worked as physical diffusion barriers and did not obviously improve electrochemical passivation or film compactness. Furthermore, the small value of the CPE exponent (*n* = 0.39789 for the film element) showed a considerable surface heterogeneity and structural imperfections of the protective layer that facilitated the penetration of the electrolyte and decreased the stability of the film. As a result, the SiO_2_-containing system offered worse corrosion protection than the TiO_2_-containing formulation^41,42^.

The difference in equivalent circuits, as seen in Fig. 8c, is related to the evolution of the corrosion protection mechanism with increased MBT concentration and the integration of nanoparticles. The blank, 0.5 MBT and 1 MBT systems showed a Warburg element (Wo), meaning a particularly porous structure of the protective layers and that diffusion processes affected the corrosion behavior through penetration of the electrolyte and transport of the chloride ions.

For the higher concentrations of MBT (1.5 MBT and 2 MBT), the Warburg element was not observed because MBT formed a more compact and continuous protective film that effectively hinders the diffusion of ions, and the corrosion mechanism shifts to be predominantly film-controlled and charge transfer-controlled.

However, the Warburg element showed up again following the inclusion of nanoparticles (2 MBT–TiO_2_ and 2 MBT–SiO_2_) because of the development of more complex nano-hybrid multilayer coatings. Diffusion in these systems proceeded through the nano-scale pathways within the hybrid film rather than by direct penetration to the metal surface. The TiO_2_-containing system displayed very limited diffusion due to the dense semiconducting TiO_2_/MBT network, while the SiO_2_-containing system showed diffusion associated with a more heterogeneous and porous insulating layer. Thus, the circuit evolution reveals that the addition of the nanoparticles has significantly altered both the film architecture and the major electrochemical transport mechanism.

**Table 3:** The EIS fitting plots for all systems were used to obtain the reference parameters.

System	Blank	0.5 MBT	1 MBT	1.5 MBT	2 MBT	2 MBT- TiO_2_	2 MBT- SiO_2_
R_s,_ (Ω cm²)	13.82	57.3	19.99	34.84	68.64	38.4	37.88
Error%	1.6	4.5	2.08	4.04	3.2	2.2	1.2
R_f,_ (Ω cm²)	–	1042	1233	7974	14,168	66,614	871.5
Error%	–	14.9	9.5	6.1	10	6.8	2.2
CPE-T (Q), (Ω^−1^s^n^cm^− 2^)	4.163 × 10^− 5^	2.9 × 10^− 5^	1.8 × 10^− 6^	1.79 × 10^− 7^	2.1 × 10^− 5^	5.0638 × 10^− 7^	9.12 × 10^− 7^
Error%	13.0	12.8	12.0	11.1	10.08	9.08	20.7
CPE-p (n)	0.96845	0.89624	0.9963	0.999	0.7788	0.9	0.84352
Error%	10.7	11.6	0.6	5.6	5.3	9.1	5.3
τ_ct, (s)_	2.56 × 10^− 2^	2.02 × 10^− 2^	2.17 × 10^− 3^	1.54 × 10^− 2^	2.11 × 10^− 1^	2.31 × 10^− 2^	2.11 × 10^− 2^
	0.02	0.03	0.009	0.004	0.08	0.004	0.001
R_ct,_ (Ω cm²)	690.4	200	1133	2321	7658	4000	1120
Error%	6.8	12.8	13.05	13.04	15.09	5.9	15.8
CPE-T (Q), (Ω^−1^.s^n^.cm^− 2^)	–	6.026 × 10^− 7^	1.5 × 10^− 6^	1.062 × 10^− 6^	3.56 × 10^− 6^	1.3171 × 10^− 6^	8.7993 × 10^− 5^
Error%	–	20.0	3.09	7.34	11.8	9.08	9.7
CPE-p (n)	–	0.899	0.99	0.9529	0.8778	0.9	0.39789
Error%	–	0.5	0.8	2.1	1.4	1.0	0.6
τ_f, (s)_	–	2.35 × 10^− 4^	2.07 × 10^− 3^	6.41 × 10^− 3^	4.24 × 10^− 3^	2.94 × 10^− 3^	2.96 × 10^− 3^
Error%		0.001	0.003	0.06	0.009	0.008	0.05
Wo-R, (Ω cm^2^)	1553	9785	31,875	–	–	82,225	54,177
Error%	6.3	14.1	19.1	–	–	6.9	9.8
Wo-T, (s)	25.72	96.35	40.74	–	–	44.89	43.9
Error%	6.09	14.8	7.9	–	–	9.02	6.9
Wo-p	0.16606	0.31177	0.25229	–	–	0.3141	0.478
Error%	10	15.09	9.7	–	–	8.40	0.6
Chi-squared	0.044998	0.031857	0.014563	0.021821	0.014917	0.015121	0.01061

Relaxation time constants (τ) were derived from Eq. 7^27^.

7 $${\mathrm{~}}\tau ={\mathrm{~}}{\left( {{\mathrm{R~}} \times {\mathrm{~Q}}} \right)^{1/{\mathrm{n}}}}$$

where R is the resistance element, Q is the constant of CPE, and n is the exponent of CPE. Higher values of τ indicate slower electrochemical kinetics and higher protection against corrosion^27^.

The relatively large value of τ_ct_ for the 2 MBT system indicates very slow charge transfer kinetics due to the very high R_ct_ value. But it is not right to judge the entire protection efficacy only by τ. Stability, homogeneity, and diffusion resistance of the film are all important variables. The 2 MBT–TiO_2_ system is the best overall formulation, showing simultaneously the highest R_ct_, the largest diffusion resistance, a very good film resistance and very homogeneous capacitive behavior. On the other hand, the system 2 MBT–SiO_2_ showed the smallest τ_ct_ value, which confirmed the quick electrochemical activity and low inhibition efficiency, although there was a diffusion barrier effect. Relaxation times (τ) were calculated in (Table 3) for both two-time-constant^27^.

where τ_ct_ and τ_f_ indicate whether the corrosion protection is regulated by charge transfer resistance or film barrier characteristics.

The EIS analysis showed that overall, MBT acts as an efficient corrosion inhibitor for brass in chloride-containing environments through adsorption and formation of a protective film^14^. TiO_2_ nanoparticles showed a strong synergistic effect, leading to a significant improvement in the film compactness, electrochemical stability and diffusion resistance. Therefore, the 2 MBT–TiO_2_ hybrid system displayed the best electrochemical performance and the highest long-term corrosion protection efficacy among all the systems studied^17,18,41^.

**Fig. 9:**
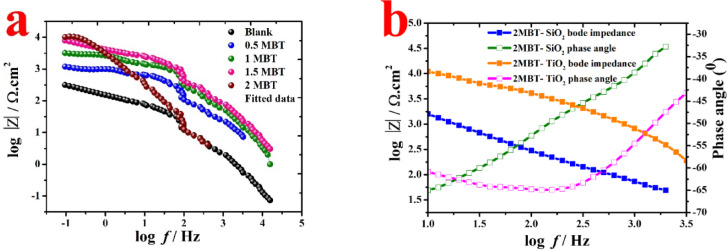
Bode plots for brass measured in 3.5% NaCl solution in the absence and presence of different concentrations of the studied inhibitors (**a**) MBT, and (**b**) 2 MBT- nano systems.

The Bode plots (Fig. 9a, b) support the Nyquist analysis. The inhibited systems presented larger impedance modulus values and more extended phase angle peaks compared to the blank solution, which confirms the improved capacitive behavior and better corrosion resistance. The 2 MBT–TiO_2_ system presented the highest impedance response and the largest phase angle maximum^56^, which indicates the production of the most homogeneous and highly capacitive protective layer^17,27^. On the contrary, the system with SiO_2_ had lower impedance values and unstable capacitive behavior, indicating weaker protection efficiency.

### EIS response recorded at the stabilized OCP

**Fig. 10:**
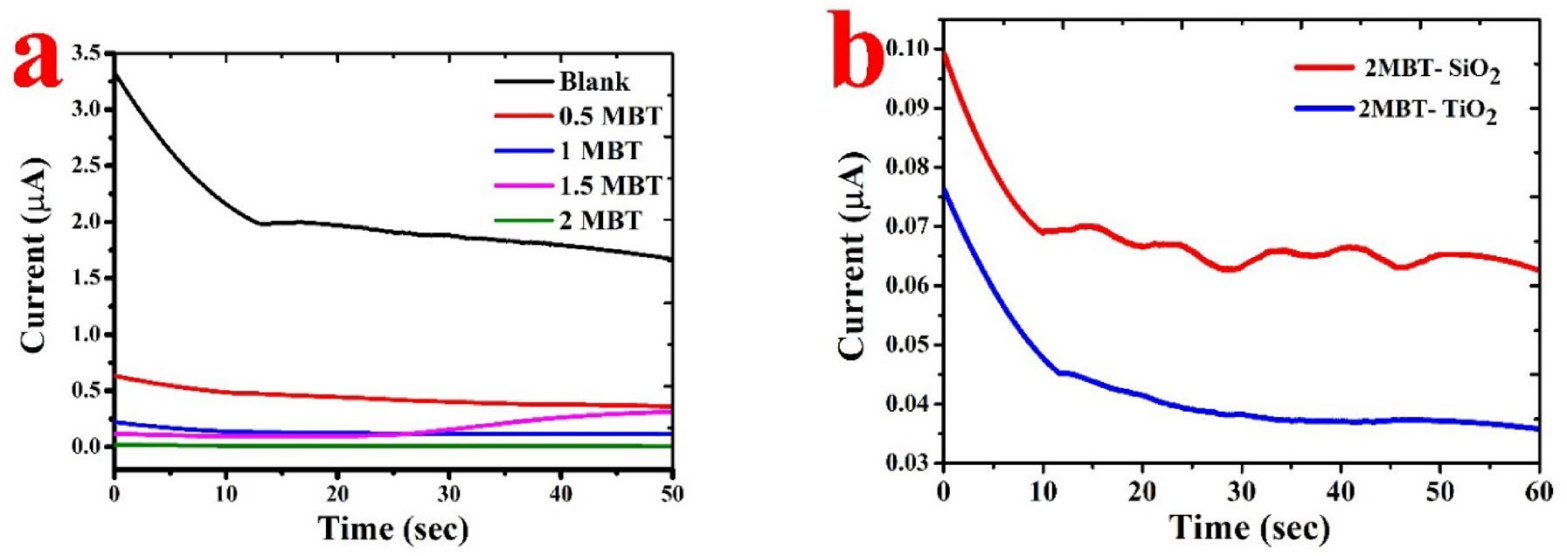
Electrochemical impedance response of brass over the investigated frequency range (**a**) blank, MBT with different concentrations, and (**b**) nanoparticles SiO_2_ and TiO_2_ addition samples of the studied inhibitors for Brass alloy in 3.5% NaCl.

Figure 10a and b present the electrochemical impedance response of brass at the stabilized open circuit potential (OCP) for frequencies ranging from 0.1 Hz to 100 kHz. The results clearly show a significant improvement in corrosion resistance with the addition of MBT^14^, and further improvement is shown in the presence of nanoparticles^17,18,21,24^. The increase in impedance response suggests stronger resistance to charge transfer at the metal/electrolyte contact, verifying the efficient adsorption of MBT molecules on the brass surface. This absorption results in the formation of a protective barrier layer, which decreases the interaction between the corrosive medium and the metal surface with increasing MBT concentration. The existence of nanoparticles further reinforces this protective barrier by enhancing its compactness and stability, inducing a more remarkable inhibitory effect, especially in the MBT-TiO_2_ system^41^. This behavior points to the synergistic interaction of MBT with nanoparticles^27^, which results in higher surface coverage and corrosion prevention properties. The impedance behavior reveals that the concentration of MBT, as well as the addition of nanoparticles of different types (insulating Vs conducting), play a vital role in modulating the electrochemical activity of the brass surface and improving its corrosion resistance over a period of time. Different MBT concentrations and the inclusion of nanoparticles were investigated for further optimization of the corrosion inhibition efficacy for possible applications^57^. The observed behavior correlated with an increase in the electrochemical time constant, suggesting slower charge transfer kinetics, as seen in Fig. 10b.

### The inhibition efficiency

From the corrosion characteristics (Table 2), the inhibition efficiency percentage for Brass alloy in 3.5% NaCl can be computed using Eqs. 8,

8 $$\eta \% {\text{ }}=\frac{{{\mathrm{C}}.{\mathrm{R}}. - {\mathrm{~C}}.{\mathrm{R}}{._{\mathrm{i}}}.{\mathrm{~}}}}{{{\mathrm{C}}.{\mathrm{R}}.}} \times 100$$

where C.R. and C.R._i_ represent the corrosion rates of brass after and before exposure to different doses of MBT inhibitors and nanoparticles, respectively^58^.

**Fig. 11:**
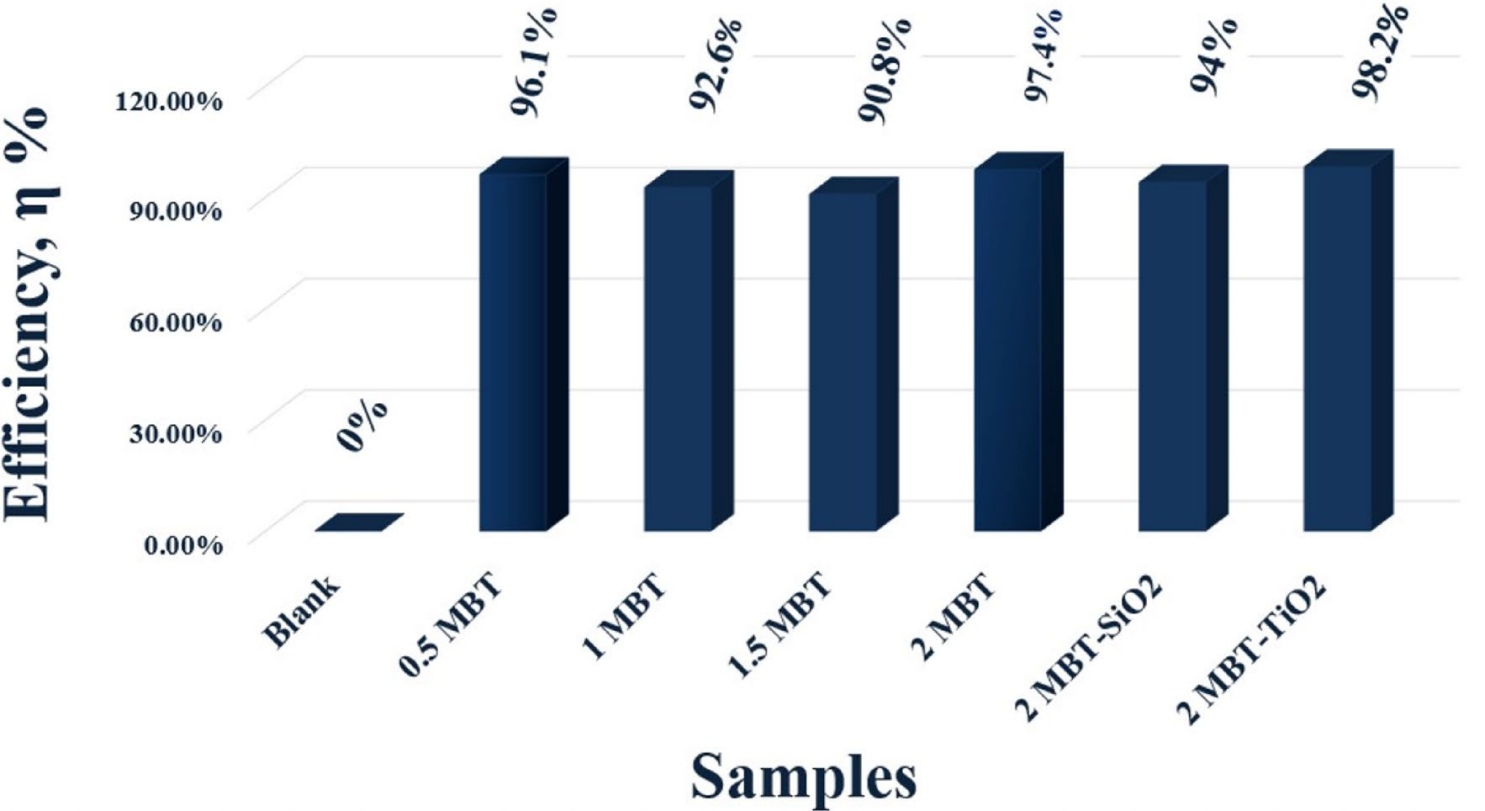
Variation of the inhibition efficiency with the concentration of the studied inhibitors for brass alloy in 3.5% NaCl.

MBT inhibition efficacy increased with concentration from 0 to 2%, as demonstrated in Fig. 10^37^. The inclusion of TiO_2_ can provide a novel, outstanding system with exceptional corrosion resistance and a 98.2% efficiency rate in delaying the reaction to environmental stimuli, acting as mixed-type inhibitors, whereas SiO_2_ has a 94% efficiency rate. At this concentration, the brass surface is covered by a greater number of inhibitor molecules, as seen in Fig. 11.

### Surface analysis and corrosion mechanism

#### SEM and EDS analysis

**Fig. 12:**
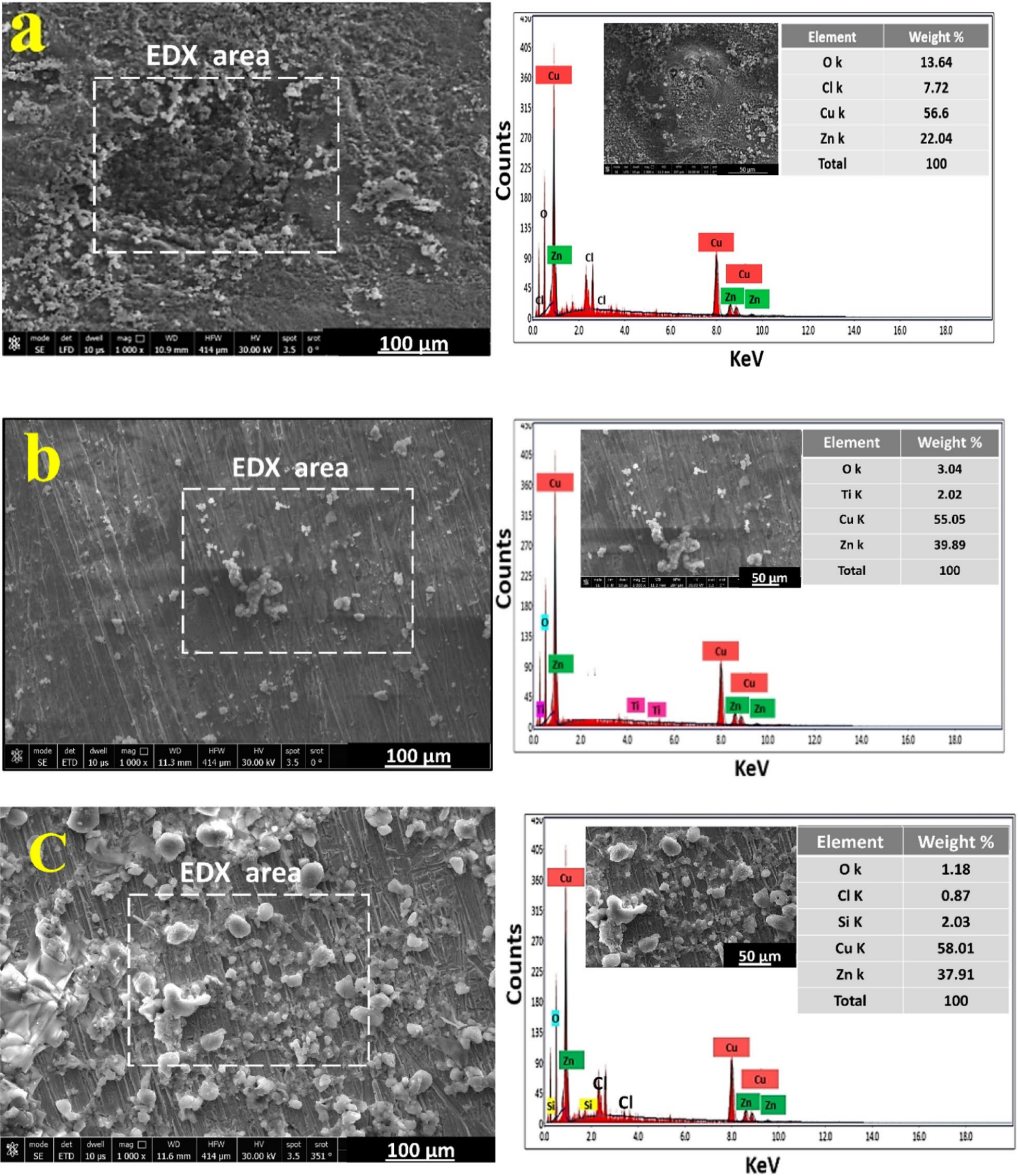
(**a**–**c**) SEM images and EDX analysis of the samples (**a**) blank, (**b**) 2MBT-TiO_2_, and (**c**) 2MBT-SiO_2_ after corrosion.

SEM images obtained after the corrosion test (Fig. 12) illustrate the surface degradation and the efficiency of the inhibitor systems applied. The aggressiveness of the chloride medium is proven by the significant damage caused by corrosion on the unmanaged brass surface^24^, presenting irregular pits, cracks and numerous corrosion products as is seen in Fig. 12a. The related EDX spectrum shows characteristic peaks of oxygen and chlorine, which suggest the production of oxide layers and chloride-containing corrosion products, which are typical of brass corrosion in NaCl conditions^46^.

Introduction of MBT showed considerable improvement in surface morphology. It indicates the formation of a protective adsorbed film, which partially protects the metal surface from the corrosive environment, as the surface becomes relatively smoother with fewer defects. This is further corroborated by a reduction in the strength of the chlorine signal in EDX research, which suggests that the chloride attack has been preserved under control. From Fig. 12b, it is clear that the inclusion of TiO_2_ nanoparticles^59,60^ leads to a significant improvement. The SEM image indicates the formation of a dense and adherent protective layer because of the presence of a surface practically free of defects, homogeneous and very compact. The EDX results show that the MBT–TiO_2_ system has a lower number of corrosive elements, which means it has higher barrier performance^61,62^.

On the other hand, as shown in Fig. 12c, the surface of the MBT-SiO_2_ system is relatively rough with visible cracks and micro-defects, indicating the surface coverage is not full, and the protection efficiency is low. In this case, the EDX spectrum still exhibits the presence of corrosion-related elements, which indicates SiO_2_ provides less protection than TiO_2_.

The surface measurements are in good agreement with the results of the adsorption isotherm, which reveals the formation of a dense and uniform protective layer as per the Langmuir adsorption behavior^27,29^.

#### Proposed inhibition mechanism

**Fig. 13:**
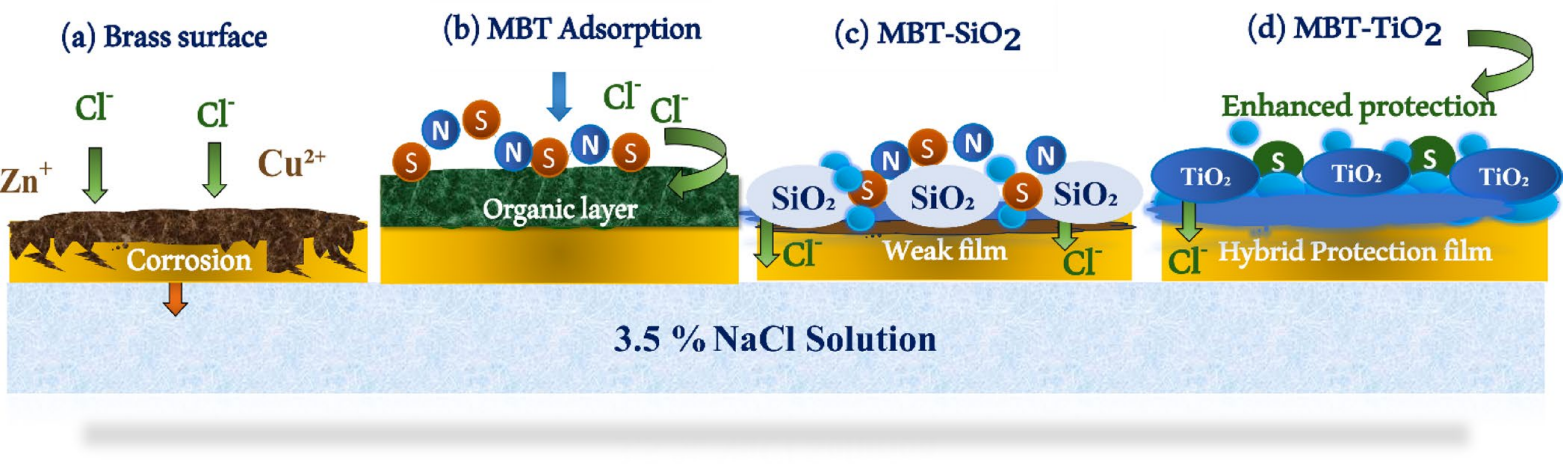
Corrosion inhibition mechanism of the MBT-based system with the presence of TiO_2_/SiO_2_ nanoparticles on the brass surface in 3.5% NaCl solution (Schematic illustration prepared using Microsoft PowerPoint software).

The corrosion inhibition mechanism of the studied systems may be seen as a progressive interfacial protection process where both molecular adsorption and nanoparticle-assisted film stabilization collaborate in suppressing the electrochemical deterioration of brass in NaCl medium^63^. As depicted in Fig. 13b, the inhibitory mechanism begins with the adsorption of MBT molecules onto the brass surface, driven by electron-rich heteroatoms, predominantly S and N. These active centers have a strong affinity for the free orbitals of the metal surface, which enables the creation of an adsorbed protective film, thereby effectively separating the active corrosion sites from the aggressive chloride environment^64^.

Thermodynamic adsorption parameters evidenced that the process is governed by a mixed physisorption–chemisorption mechanism, indicating that MBT molecules are not only physically accumulated at the interface but also strongly anchored to the metallic surface by coordinated electronic interactions^14^. Therefore, the created layer is the first electrochemical barrier to charge transfer reactions and to the entrance of chloride, as seen in Fig. 13b.

However, it was revealed that the protective efficacy of the hybrid systems was greatly dependent on the composition of the included nanoparticles. Still, both TiO_2_ and SiO_2_ nanoparticles contribute to the enhancement of the barrier properties of the adsorbed layer; their effects on the electrochemical interface are fundamentally different due to their varied electronic properties^65^.

In the SiO_2_-containing system, as seen in Fig. 13c, the nanoparticles are mostly inert insulating fillers. Their function is essentially restricted to the extension of the diffusion path of corrosive ions and the partial reduction of the permeability of the electrolyte. However, this physical barrier contribution does not prohibit SiO_2_ from having relatively modest electrical interaction with both the brass surface and MBT molecules^61^. Thus, the resulting protective layer is rather heterogeneous and porous, leading to local penetration of electrolyte and limiting the stability of the electrochemical interface. This explains the relatively moderate corrosion resistance and the preservation of the diffusion-controlled behavior exhibited on the impedance spectra.

In comparison, the TiO_2_-containing system showed quite distinct behavior, as seen in Fig. 13d. TiO_2_ is a semiconducting oxide with increased surface activity and stronger interfacial electronic interaction with MBT molecules and brass substrate^65^. This semiconducting character helps in charge redistribution at the inhibitor/metal interface and improves the adsorption and the anchoring of the MBT species on the metal surface. Consequently, the formation of a more compact, uniform and defect-free protective coating occurs. The synergistic effect of MBT and TiO_2_ can effectively inhibit the charge transfer reaction and the ionic diffusion process, resulting in a notable improvement in the electrochemical stability^65^. This method is further confirmed by SEM and EDX data in Fig. 12.

Electrochemical tests clearly corroborate this interpretation. The system containing TiO_2_ displayed considerably greater charge transfer resistance (R_ct_) values, combined with better capacitive properties, than the system containing SiO_2_. The enhanced capacitive response implies that a thicker and more stable interfacial layer is formed, which can provide long-term protection against chloride attack.

Therefore, the present work reveals that corrosion protection in hybrid inhibitor systems depends not only on the physical barrier effect of nanoparticles but also on their inherent electronic properties and interfacial activity^66^. The semiconducting TiO_2_ nanoparticles promote a synergic electrochemical inhibition process with MBT molecules by enhancement of adsorption, interfacial charge stability and film densification. Meanwhile, the contribution of the SiO_2_ nanoparticles is predominantly by passive diffusion blocking with low electrochemical engagement^61^.

Finally, the inhibition mechanism can thus be summarized as a cooperative process, where MBT molecules adsorb on the brass surface through the S and N active centers to form the main protective layer^66^, while TiO_2_ nanoparticles reinforce the protective layer by improving its compactness, structural integrity and electrochemical stability, showing better long-term corrosion resistance in chloride medium.

### Quantum chemical calculations

The molecular structures and geometrical factors were investigated to better understand the electrical behavior and stability of the analyzed systems, which were derived by density functional theory (DFT)^29^.

The binding energy per atom was also calculated to evaluate the relative stability and interaction intensity within each system.

The relative stability of the systems was assessed by calculating the binding energy per atom (B.E.). This value gives an understanding of the strength of atomic interactions and overall structural stability. A lower number of $\:{E}_{B}$ indicates a more stable system. Equation (9) calculated the binding energy per atom^27^.

9 $$~{E_B}=\frac{{{E_s} - \sum \left( {{N_E} \cdot {E_E}} \right)}}{{{N_s}}}~$$

where$\:{E}_{s}$ is the total energy of the system, $\:{N}_{E\:}$ is the number of atoms of each element, $\:{E}_{E}$ is the corresponding atomic energy of each element and $\:{N}_{s\:}$ is the total number of atoms in the system. This formulation enables a consistent evaluation of stability between different configurations, regardless of their size or composition.

#### Descriptors in electronic structure and reactivity

The electronic characteristics of the studied systems were discussed using frontier molecular orbital (FMO) theory, in which the highest occupied molecular orbital (HOMO) and lowest unoccupied molecular orbital (LUMO) energies play a key role in molecular reactivity. According to Koopmans’ theorem^67,68^. The ionization potential (I) and electron affinity (A) can be approximated by the negative of the HOMO and LUMO energies, respectively. The energy gap ($\:{E}_{\mathrm{g}\mathrm{a}\mathrm{p}}$), which is the difference between LUMO and HOMO energy, is an important parameter of chemical reactivity and stability, where smaller values indicate more reactivity, as expressed in Eq. (10).

10 $$~{E_{{\mathrm{gap}}}}{\mathrm{~}}={E_{LUMO}} - {E_{HOMO}}~$$

Accordingly, the ionization potential and electron affinity are defined as given in Eqs. (11) and (12):

11 $$I= - {E_{HOMO}}$$

12 $$A= - {E_{LUMO}}~$$

Based on these quantities, the chemical potential ($\:\mu\:$), which reflects the tendency of electrons to escape from the system, is calculated in Eq. (13).

13 $$~\mu = - \frac{{I+{\mathrm{A}}}}{2}$$

The global hardness ($\:\eta\:$), describing resistance to charge transfer, and the corresponding softness ($\:S$), representing the ease of electron cloud deformation, are given in Eqs. (14) and (15):

14 $$~\eta \approx \left( {E_{{LUMO}} - E_{{HOMO}} } \right)/2$$

15 $$~S=1/2\eta$$

Furthermore, the electrophilicity index (ω), which measures the stabilization energy upon acquiring additional electronic charge, is expressed as Eq. (16):

16 $$\omega ={\mu ^2}/2\eta$$

In addition, the maximum charge transfer $\:\left(\varDelta\:\:{N}_{max}\right)$ between the inhibitor and the metal surface is estimated using Eq. (17):

17 $$\Delta N_{{max}} = ~{{ - \mu } \mathord{\left/ {\vphantom {{ - \mu } \eta }} \right. \kern-\nulldelimiterspace} \eta }$$

The global reactivity descriptors give a full understanding of the electrical behavior of the examined systems in terms of their stability, reactivity and electron-donating and accepting capacity. Such parameters are especially significant for the evaluation of the adsorption strength and corrosion inhibition performance at the molecular level^69–73^.

#### Adsorption energy and analysis

Adsorption energy estimates were also performed to better understand the interaction intensity between MBT and nanoparticle-modified surfaces.

The interaction strength between MBT and the nanoparticle-modified systems was evaluated by calculating the adsorption energy ($\:{E}_{ads}$). The adsorption energy can be calculated by Eq. (18)^27^:

18 $$~{E_{ads}}=~{E_{complex}} - \left( {{E_{surface}}+{E_{inhibitor}}} \right)~$$

where $\:{E}_{complex}$, $\:{E}_{surface}$ and $\:{E}_{inhibitor}$ are the total energies of the adsorbed system, the surface and the isolated inhibitor molecule, respectively. Negative $\:{E}_{ads}$ values are more indicative of the stronger adsorption and greater stability of the generated complex^74,75^.

The estimations of the adsorption energy are in agreement with the electronic analysis and experimental electrochemical results. The ($\:{E}_{ads}$) of MBT–TiO_2_ (−2.606 eV) is more negative than that of MBT–SiO_2_ (−1.976 eV), indicating the creation of a more stable and adsorbed protective layer. This correlates well with the increase in the charge transfer resistance and inhibitory efficiency, which was achieved experimentally. The negative value of ΔG°_ads_ (−12.66 kJ mol^− 1^) indicates that the adsorption of MBT on the brass surface is spontaneous and stable, which is in agreement with DFT results. Therefore, the excellent corrosion protection properties of TiO_2_ are attributed not only to the barrier effect but also to the capacity to improve the interfacial electronic coupling and to stabilize the adsorption inhibitor coating on the metal surface^74–77^.

#### Electronic structure and frontier molecular orbital (HOMO–LUMO)

**Fig. 14:**
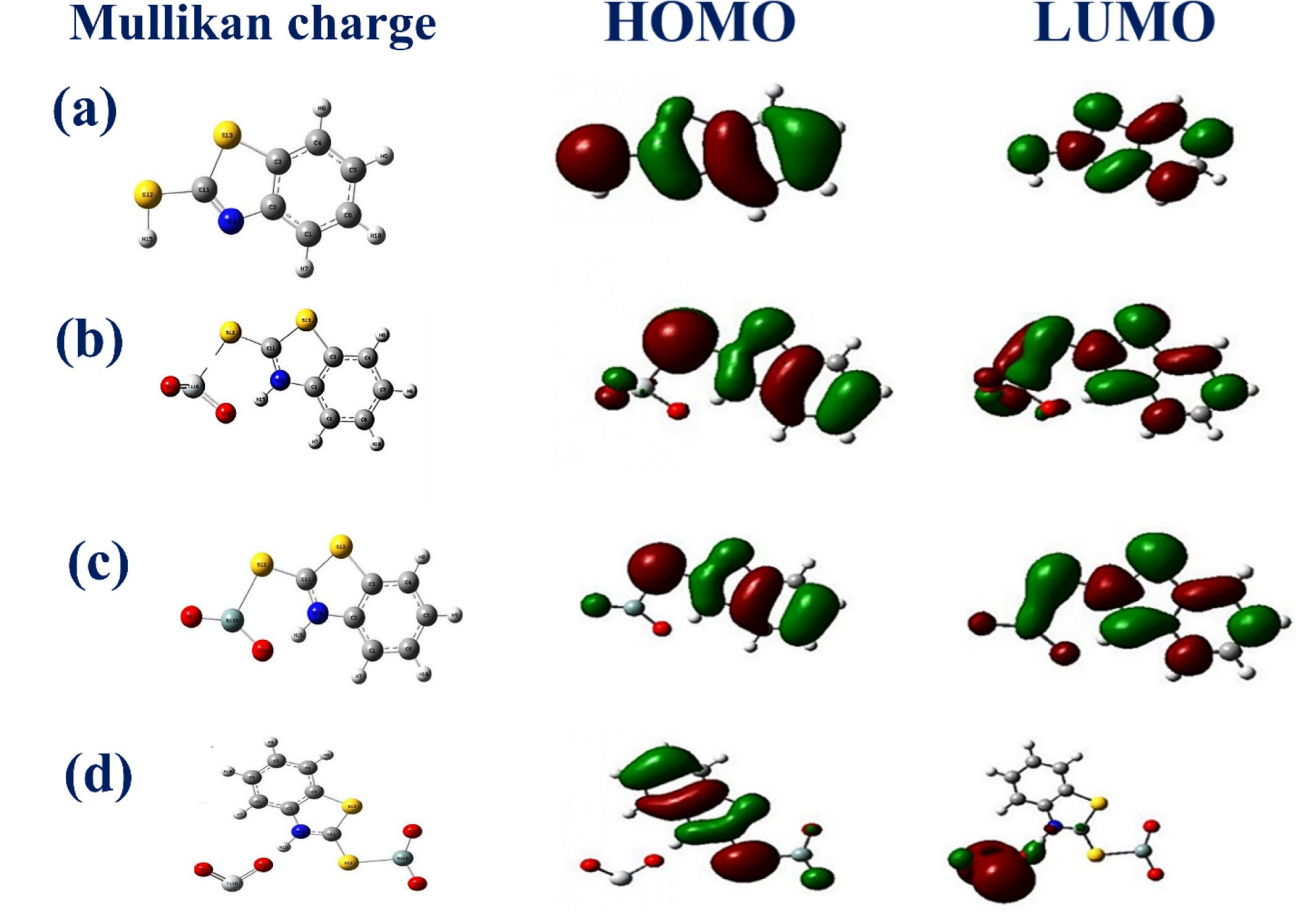
Molecular structures of some major constituents of the MBT-nano system (**a**) 2 MBT, (**b**) 2 MBT-TiO_2_, (**c**) 2 MBT-SiO_2_, and (**d**) 2 MBT-TiO_2_-SiO_2_.

Figure 14 displays the optimized molecular structures and frontier molecular orbital distributions of the studied systems. The inherent geometry of the solitary MBT molecule remains unchanged (Fig. 14a), but considerable electronic redistribution is observed following interaction with TiO_2_ and SiO_2_ nanoparticles. In the MBT–TiO_2_ system (Fig. 14b), the interaction occurs predominantly at the sulfur and nitrogen active centers, exhibiting strong interfacial coordination and rapid charge transfer. On the contrary, for the MBT-SiO_2_ combination (Fig. 14c), the orbital localization and interfacial interaction are weaker because of the insulating property of SiO_2_. The combined MBT-TiO_2_-SiO_2_ system (Fig. 14d) leads to a more complicated and stabilized adsorption structure, showing the influence of the oxide surface chemistry on the molecular organization and electronic distribution of the MBT.

The computed electronic parameters also support these observations. The embedded nanoparticles decrease the energy gap $\:{(E}_{\mathrm{g}\mathrm{a}\mathrm{p}})$ compared to the isolated MBT, which indicates the higher electronic activity and tendency of adsorption. The lowest energy gap (4.5427 eV) is obtained for MBT–TiO_2_ among all the configurations, which suggests the highest chemical reactivity and most potent electron exchange ability with the metal surface. The MBT–SiO_2_ has a relatively larger energy gap (4.7746 eV), indicating the weak electronic interaction and adsorption behavior. Moreover, the binding energy measurements demonstrated that the MBT–TiO_2_ system had the maximum structural stability and the most thermodynamically favorable adsorption configuration.

The Frontier Molecular Orbital (FMO) theory has been extensively used to understand the electrical reactivity and adsorption properties of corrosion inhibitors^27–29^. The HOMO energy is a measure of the electron-donating ability of the inhibitor, while the LUMO energy is a measure of the electron-accepting tendency^76,77^. These results are further supported by the distribution of HOMO-LUMO as shown in Fig. 14. For the MBT-TiO_2_ system, the HOMO and LUMO concentrations are well confined at the adsorption interface and around the heteroatoms (S and N), which allows efficient processes of electron donation and acceptance. Such localization indicates substantial orbital overlapping and better interfacial charge transfer. In contrast, the MBT–SiO_2_ orbital distributions are more delocalized with less electron density accumulation at the adsorption centers, which is in good agreement with the weaker adsorption interaction.

#### Global reactivity descriptors

The calculated global reactivity descriptors, such as electron affinity ($\:A$), ionization potential ($\:I$), chemical potential (µ), chemical hardness (η), softness (S) and electrophilicity index (ω), provide a clear picture about the electronic behavior of the studied systems, as given in Table 4. The integration of nanoparticles enhances electron affinity, yielding higher values in the modified systems (2.035 eV for MBT–TiO_2_, 2.4056 eV for MBT–SiO_2_, and 2.3237 eV for MBT–TiO_2_–SiO_2_) than in pristine MBT (1.4751 eV), indicating greater electron-accepting ability. Correspondingly, the ionization potential increases in the modified systems, indicating a larger barrier to electron loss and improved electrical stability^27–29^.

The alteration leads to a larger negative chemical potential (µ), especially in the case of MBT–SiO_2_ (− 4.7929 eV), indicating a higher tendency for the acceptance of electrons. On the contrary, the chemical hardness (η) reduces especially for MBT–TiO_2_ (2.27135 eV) while the chemical softness (S) improves, which confirms the improved polarizability and charge transfer capabilities between MBT molecules and TiO_2_.

Moreover, the electrophilicity index (ω) increases for all changed systems and attains its maximum value for MBT–SiO_2_ (4.81127 eV), followed by MBT–TiO_2_–SiO_2_ and MBT–TiO_2_. This suggests a higher electron-accepting nature of the changed systems, but this does not mean that it correlates with the adsorption stability in all circumstances.

Overall, the computed adsorption energies and experimental inhibition results agree with the trends in the global reactivity descriptors. Particularly for MBT–TiO_2_, the decrease in chemical hardness (η) and rise in softness (S) indicate better polarizability and charge transfer capabilities, which encourage the creation of a more stable adsorbed layer. This is in good agreement with the greater adsorption energy found from DFT simulations. Adsorption stability is primarily controlled by electron donation, back-donation, and interfacial orbital interaction rather than only ω, even though the electrophilicity index (ω) rises for the changed systems. As a result, the MBT–TiO_2_ system has the best electrical characteristics and the best adsorption performance, which is consistent with both theoretical and experimental results.

#### Molecular electrostatic potential

**Fig. 15:**
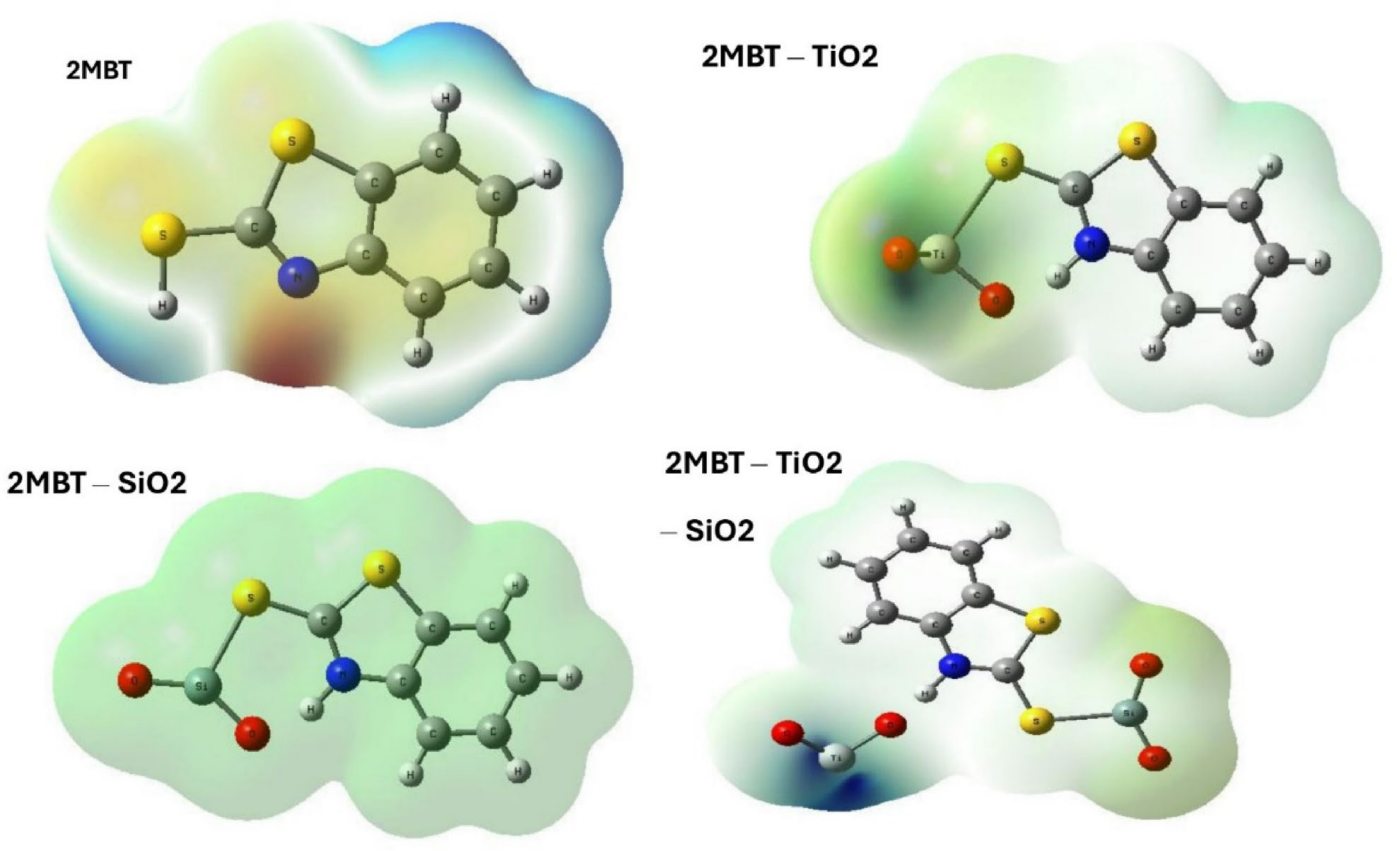
Molecular electrostatic potential (MEP) surfaces calculated at the B3LYP/LANL2DZ level of theory.

The MEP surfaces provide us with useful information on the charge distribution and reactive sites of the researched systems. As shown in Fig. 15, the electron-rich areas (red zones) are predominantly around the sulfur and nitrogen atoms of MBT, which is consistent with the main adsorption locations.

The adsorption interface reveals a remarkable enhancement of the negative electrostatic potential upon interaction with TiO_2_ nanoparticles, which points to increased electron density and robust charge transfer between the inhibitor and the oxide surface. This behavior indicates significant interfacial contact and supports the creation of a stable adsorption complex.

However, the electron distribution is more diffuse in the MBT–SiO_2_ system, indicating weaker electronic interaction due to the more inert nature of SiO_2_. In the MBT–TiO_2_–SiO_2_ system, there are several reactive areas with improved polarization, but the TiO_2_ still has the primary contribution.

The total MEP findings are in agreement with the adsorption energy and frontier molecular orbital analysis results, which confirmed that the addition of TiO_2_ greatly boosts the adsorption strength and corrosion inhibition efficacy of MBT^75^.

#### Dipole moment and charge transfer

The dipole moment is greatly increased after modification, with the largest for MBT–TiO_2_–SiO_2_ (22.889 Debye), followed by MBT–SiO_2_ (14.291 Debye) and MBT–TiO_2_ (9.5386 Debye) in comparison to MBT (1.9757 Debye), suggesting the higher polarity and adsorption propensity.

Moreover, all changed systems exhibit an increase in ($\:\varDelta\:\:{N}_{max}$), implying better adsorption with the metal surface^29^. The improvements of both dipole moment and ($\:\varDelta\:\:{N}_{max}$) generally support the increased adsorption strength and better inhibitory performance of the modified systems, which makes it an excellent means of inhibition and protection, as seen in Table 4.

**Table 4:** The calculated quantum parameters of the structures at B3LYP/LANL2DZ basis set in a 3.5% NaCl aqueous solution.

System	B.E. (eV)	E_HOMO_ (eV)	E_LUMO_ (eV)	$\:{E}_{\mathrm{g}\mathrm{a}\mathrm{p}}$ (eV)	A (eV)	I (eV)	µ (eV)	η (eV)	S (eV^− 1^)	$\:\omega\:$ (eV)	ΔN_max_ (eV)	Dipole moment (Debye)
2MBT	− 5.7920	− 6.6501	− 1.4751	5.175	1.4751	6.6501	− 4.0626	2.5875	0.38647	3.1893	1.57009	1.975
2MBT-TiO_2_	− 6.1604	− 6.5777	− 2.0350	4.5427	2.035	6.5777	4.30635	2.27135	0.440267	4.082297	1.89594	9.5386
2MBT-SiO_2_	− 5.8989	− 7.1802	− 2.4056	4.7746	2.4056	7.1802	− 4.7929	2.3873	0.418883	4.81127	2.00767	14.291
2MBT-TiO_2_-SiO_2_	− 6.1188	− 6.8999	− 2.3237	4.5762	2.3237	6.8999	− 4.6118	2.2881	0.437044	4.647677	2.01556	22.889

Finally, the combined electrochemical, surface and theoretical study revealed that the MBT-nano systems considerably enhance the corrosion resistance of brass in NaCl conditions. The inhibitory effect was mainly related to the adsorption of MBT molecules and the creation of a dense protective film that prevented the permeation of destructive chloride ions. The addition of nanoparticles led to an improvement in the stability and surface coverage of the protective layer through synergistic interaction with MBT molecules. Also, the DFT calculations were in good agreement with the experimental data, which corroborate the strong adsorption and inhibition efficiency of the studied systems. Among all the systems studied, the combination of MBT-TiO_2_ showed the best corrosion protection performance, due to its increased capacity to build a durable and highly resistant barrier coating in 3.5% NaCl solution.

**Table 5:** Comparison of previously reported corrosion inhibitors with the present work.

System	Medium	Conc., (ppm)	η %	Adsorption behavior	Mechanism insight	References
Heterocyclic inhibitors on a variety of metals and alloys	HCl/H_2_SO_4_	5–50 ppm	88–95.0%	Mixed	π-electron interaction	^4^
MBT+Tween-80 on brass	0.2 M NaCl	Optimum Conc.	94.0%	Mixed-type inhibition with Langmuir adsorption behavior	Synergistic adsorption enhanced surface coverage and suppressed dezincification of brass	^13^
MBT alone on brass	Sulfide-polluted 3.5% NaCl	1 × 10^− 6^ -1 × 10^− 3^ M MBT + 100–1000 ppm sulfide	Reduced at high sulfide concentration	MBT protection weakened in the presence of sulfide ions	Cu–MBT protective complex was replaced by Cu_2_S films	^14^
TTA + SiO_2_/ TiO_2_ NPs on brass	3.5%NaCl	TTA 0.5ppm NPs 10 ppm	SiO_2_ 99.03% TiO_2_ 98.1%	Physisorption	Physical barrier + nano filler	^15^
MBTA on brass	3%NaCl	150 ppm	71.0%	Mixed-type inhibitor following the Langmuir adsorption isotherm	Organic inhibitor adsorbed strongly on the brass surface and reduced the corrosion rate	^16^
BTA + Al_2_O₃ nanoparticles on brass	Simulated water/brass system	Variable NP Conc.	Decreased with increasing Al_2_O₃	Langmuir adsorption with weakened chemisorption	Al_2_O₃ nanoparticles reduced BTA adsorption and weakened the protection efficiency	^17^
Hybrid nanoparticle protective coatings on metals	Various corrosive environments	variable	–	Barrier and hybrid coating behavior	Nanoparticles improved coating durability and long-term corrosion protection	^18^
Benzotriazole derivatives on aluminum alloys	Neutral aqueous media	Variable	–	Adsorption and blocking of active sites	Corrosion inhibition occurred through adsorption and suppression of intermetallic activity	^19^
MPATA triazole derivative on brass	Neutral borate buffer + NaCl	–	High protection after salt fog exposure	Formation of Cu/Zn-organic protective nanolayer	A thin adsorbed complex layer significantly improved brass corrosion resistance	^20^
Nano-dispersoid polymer nanocomposite coatings on metals, alloys, and metallic surfaces	Polymeric coating systems	Variable	–	Smart barrier and nanocomposite behavior	Nano-dispersoids enhanced coating compactness and corrosion resistance	^21^
BTA + Sb_2_O_3_ NPs on Cu alloy	NaCl	Variable	99.96%	Physical	Barrier protection	^24^
MBT derivatives on mild steel	Acidic media	10–50 ppm	90–96%	Chemisorption	Organic inhibitor behavior	^26^
2MBT + SiO_2_ NPs (DFT supported) @ brass	3.5% NaCl	20 ppm MBT + 10 ppm SiO_2_	94.0%	Moderate adsorption behavior	Mainly, the physical barrier effect due to inert silica nanoparticles	This work
2MBT + TiO_2_ NPs (DFT supported) on brass	3.5% NaCl	20 ppm MBT + 10 ppm TiO_2_	98.2%	spontaneous adsorption dominated by physical with weak chemical interaction	Electronic interaction and charge transfer enhanced compact protective film formation	This work

As shown in Table 5, the present system outperforms previously reported MBT-based inhibitors by achieving higher inhibition efficiency at significantly lower concentrations, indicating enhanced adsorption and synergistic interaction induced by TiO_2_ nanoparticles.

## Conclusions

The combined technique of both nanoparticles, whether TiO_2_ or SiO_2_, with the ideal ratio of MBT in brass, on corrosion behavior in 3.5% NaCl solution is highlighted in this study. We discovered that:

1. 2-Mercaptobenzothiazole (MBT) interacts with metal ions with N and S atoms and accumulates on the metal’s surface, preventing corrosion of copper alloys by blocking pores in brass alloys, reducing chloride entry by complex compound production.
2. Increasing 2-MBT concentration (2%) reduces the electrochemical reaction on metallic surfaces by 97.4%, which inhibits corrosion.
3. The combination of 2-MBT inhibitor and 10 mg of TiO_2_/SiO_2_ nanoparticles in the solution has shown greater corrosion barrier performance, giving better protection under corrosive conditions and increasing their adherence, which makes the brass alloy more resistant to chemical deterioration.
4. The inclusion of TiO_2_ can create a novel, excellent synergistic inhibitor with exceptional resistance to corrosion and a 98.2% efficiency rate in delaying the response to environmental stimuli, acting as mixed-type inhibitors; in comparison, SiO_2_ has a 94% efficiency rate.
5. The synergetic effect of nanoparticles with MBT enhances the surface morphology by filling in micro-defects, leading to corrosion prevention on the brass surface.
6. The adsorption investigation revealed that the adsorption of MBT on the brass surface obeys the Langmuir adsorption isotherm model.
7. The computed adsorption equilibrium constant (K_ads_ = 2.98 Lmol^−1^) and negative free energy of adsorption (ΔG°_ads_ = − 12.66 kJ mol^−1^) exhibited spontaneous and stable adsorption of MBT on the brass surface, indicating that the adsorption process is predominantly physical in nature (physisorption), governed mainly by electrostatic interactions between the inhibitor molecules and the metal surface.
8. Polarization studies showed that MBT functions as a mixed-type inhibitor by reducing both anodic and cathodic processes.
9. The adsorption study demonstrated that the combination of physical and chemical interactions between MBT molecules and the brass surface governed the corrosion prevention mechanism.
10. Density functional theory calculations were demonstrated to be an efficient and reliable way to describe the electrical characteristics, adsorption behavior and corrosion inhibition mechanism of MBT and its modified systems.
11. A high correlation was found between the theoretical DFT parameters and the experimental electrochemical performance, proving the capabilities of DFT to characterize charge transport and interfacial interactions at the molecular level.
12. DFT simulations indicated that alteration of nanoparticles considerably increased the electrical properties and adsorption behavior of MBT.
13. The modified systems exhibited lower adsorption energy, narrower HOMO-LUMO energy gap and higher charge transfer $\:\left(\varDelta\:\:{N}_{max}\right)\:$confirming stronger adsorption affinity and better corrosion inhibition effectiveness.

## Supplementary Information

Below is the link to the electronic supplementary material.

Supplementary Material 1

## Data Availability

Data will be made available on request.
