# Supplementary material for: Superior synergistic corrosion inhibition of brass in NaCl solution by 2-mercaptobenzothiazole and TiO2 nanoparticles compared with SiO2
Source: Sci Rep. 2026 Jun 13;16:18351. doi: 10.1038/s41598-026-55234-0 (PMC13264628; doi:10.1038/s41598-026-55234-0)
Supplement: Supplementary file 1 — Supplementary Material 1 [file 41598_2026_55234_MOESM1_ESM.docx]

1. **Adsorption Isotherm Analysis Models**

The adsorption behavior of 2-mercaptobenzothiazole (MBT) on the brass surface was investigated using adsorption isotherm models in order to better understand the inhibition mechanism.

- 1. **Freundlich Adsorption Isotherm**

The surface coverage (θ) was calculated from the inhibition efficiency (η%) obtained from potentiodynamic polarization measurements using Eq. (S1) [29]:

$\theta= \frac{\eta}{100}$ (S1)

**Table S1.** Adsorption isotherm modelling data for MBT inhibitor, including concentration (ppm) and coverage angle measurements.

| **Sample** | **MBT (ppm)** | **η %** | **θ** |
| --- | --- | --- | --- |
| **0.5 MBT** | 5 | 96.1 | 0.961 |
| **1 MBT** | 10 | 92.6 | 0.926 |
| **1.5 MBT** | 15 | 90.8 | 0.908 |
| **2 MBT** | 20 | 97.4 | 0.974 |

The obtained θ values were fitted to the Freundlich Isotherm, expressed in Eq. (S2) [29]:

log θ = log *K_F_* + $\frac{1}{n}$ log C (S2)

The Freundlich adsorption isotherm was also applied to investigate the feasibility of adsorption on a heterogeneous surface. The linear version of the model (log θ vs log C) presented a very weak correlation coefficient (R^2^ = 0.009), suggesting that the adsorption of MBT molecules does not follow the Freundlich behavior. The results imply that under the analyzed conditions, the surface of brass alloy does not show substantial variability and multilayer adsorption features.

**Figure S1.** Freundlich adsorption isotherm plot (log θ vs log C) for MBT adsorption on brass surface in 3.5% NaCl solution.

1. **2. Temkin Adsorption Isotherm**

Also, the Temkin isotherm resulted in a very poor correlation (R² = 0.0078), which means that the assumption of adsorbate–adsorbate interaction does not fit for this system. Hence, the adsorption energy is independent of surface covering and the Temkin model is not relevant.

θ = *B* ln C+ *B* ln *KT* (S3)

**Figure S2.** Temkin adsorption isotherm plot (ln C vs θ) for MBT adsorption on brass surface in 3.5% NaCl solution.

- 1. **Langmuir Adsorption Isotherm**

The Langmuir isotherm showed the best fit (R^2^ =0.9941) among all concentrations studied, with a slope near unity, indicating monolayer adsorption on a homogeneous surface with minimum interaction between adsorbed species, as shown in Fig. 6. (manuscript)

The presence of nanoparticles in the adsorption modelling was neglected because they mainly operate as the film modifiers to enhance the stability and compactness of the MBT adsorbed layer instead of participating in the adsorption directly.

The Langmuir model shows the best fit (highest R²), indicating monolayer adsorption of MBT, while the Freundlich and Temkin models are not appropriate due to their very low correlation coefficients.

**Figure S3.** Langmuir adsorption isotherm plot (C_inh_ vs C_inh_/θ) for MBT adsorption on brass surface in 3.5% NaCl solution.

The obtained θ values were fitted to the Langmuir adsorption isotherm, expressed in Eq. (S4)[29]:

$\frac{C_{inh}}{\theta}= \frac{1}{K_{ads}}+C{}_{inh}$(S4)

**Table S2.** Adsorption isotherm fitting parameters and Regression Coefficients.

| **Model** | **R^2^** | **Parameters** | **Slope** | **Intercept** | **Constants** |
| --- | --- | --- | --- | --- | --- |
| **Langmuir** | 0.9941 | K_ads =_ 2.98 (L.mg^-1^) | 1.0343 | 0.0336 | --- |
| **Freundlich** | 0.009 | K_F_ = 0.953, n =-0.005 | -0.0051 | -0.0207 | Adsorption intensity |
| **Temkin** | 0.0078 | A = 0.9531, B = -0.0045 | -0.0045 | 0.9531 | Interaction parameter |
